# Pyrido[2′,1′:2,3]imidazo[4,5-*c*]isoquinolin-5-amines as Potential Cytotoxic Agents against Human Neuroblastoma

**DOI:** 10.3390/ph14080750

**Published:** 2021-07-30

**Authors:** Zahira Tber, Mohammed Loubidi, Jabrane Jouha, Ismail Hdoufane, Mümin Alper Erdogan, Luciano Saso, Güliz Armagan, Sabine Berteina-Raboin

**Affiliations:** 1 Institut de Chimie Organique et Analytique ICOA, Université d’Orléans-Pôle de Chimie, UMR CNRS 7311, Rue de Chartres-BP 6759, CEDEX 2, 45067 Orléans, France; tber_zahira@yahoo.com (Z.T.); m.loubidi@gmail.com (M.L.); jabranejouha@gmail.com (J.J.); 2 Laboratory of Molecular Chemistry, Faculty of Sciences Semlalia, Cadi Ayyad University, Marrakech BP 2390, Morocco; i.hdoufane@gmail.com; 3 Department of Physiology, School of Medicine, Izmir Katip Çelebi University, Izmir 35620, Turkey; alpero86@gmail.com; 4 Department of Physiology and Pharmacology Vittorio Erspamer, Sapienza University of Rome, P. le Aldo Moro 5, 00185 Rome, Italy; luciano.saso@uniroma1.it; 5 Department of Biochemistry, Faculty of Pharmacy, Ege University, Bornova 35100, Turkey

**Keywords:** human neuroblastoma, PARP-1 inhibitors, anticancer drugs, pyrido[2′,1′:2,3]imidazo[4,5-*c*]isoquinolin-5-amines, antiproliferative activity

## Abstract

We report herein the evaluation of various pyrido[2′,1′:2,3]imidazo[4,5-*c*]isoquinolin-5-amines as potential cytotoxic agents. These molecules were obtained by developing the multicomponent Groebke–Blackburn–Bienaymé reaction to yield various pyrido[2′,1′:2,3]imidazo[4,5-c]quinolines which are isosteres of ellipticine whose biological activities are well established. To evaluate the anticancer potential of these pyrido[2′,1′:2,3]imidazo[4,5-*c*]isoquinolin-5-amine derivatives in the human neuroblastoma cell line, the cytotoxicity was examined using the WST-1 assay after 72 h drug exposure. A clonogenic assay was used to assess the ability of treated cells to proliferate and form colonies. Protein expressions (Bax, bcl-2, cleaved caspase-3, cleaved PARP-1) were analyzed using Western blotting. The colony number decrease in cells was 50.54%, 37.88% and 27.12% following exposure to compounds **2d**, **2g** and **4b** respectively at 10 μM. We also show that treating the neuroblastoma cell line with these compounds resulted in a significant alteration in caspase-3 and PARP-1 cleavage.

## 1. Introduction

**Figure 1:**
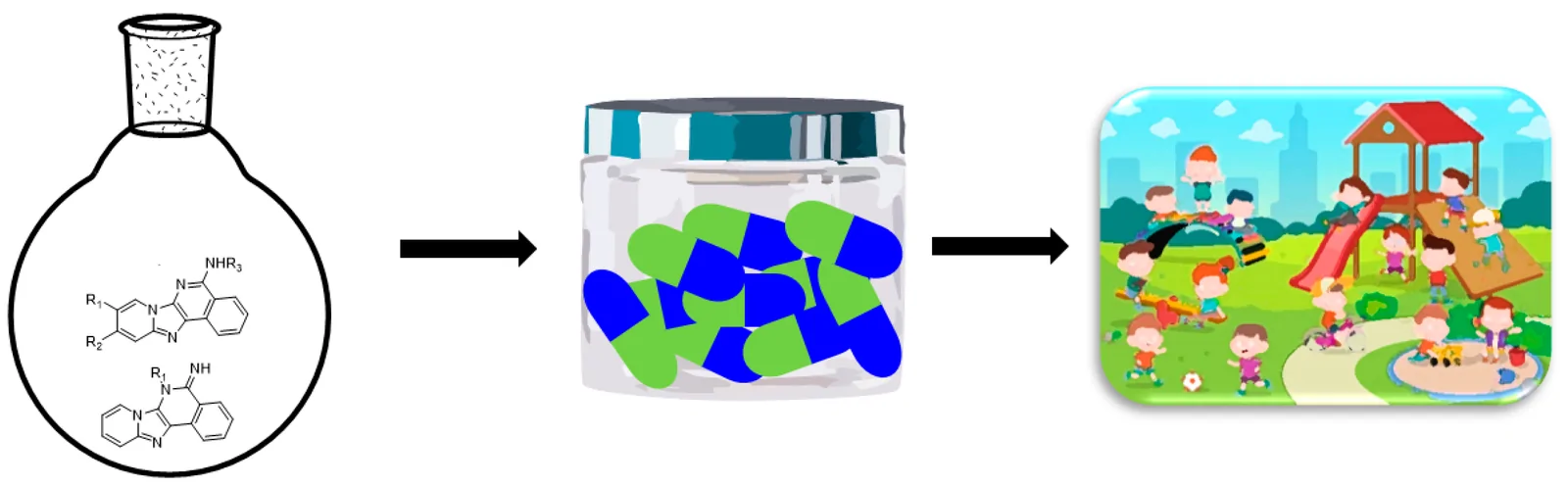
These potentially active molecules could become drug candidates for the protection of children and prevent pediatric cancer mortality.

Among the most common types of pediatric solid tumors, neuroblastoma is responsible for nearly 15% of pediatric cancer mortality [1]. Unfortunately, children at high risk show low survival rates despite rigorous treatment [1,2,3,4]. To fight against this, there are several different treatment strategies, including surgery, chemotherapy, radiation therapy, myeloablative therapy, stem cell transplantation, and immunotherapy. This treatment palette could be improved with alternative strategies including immunotherapy and novel anticancer drugs with limited adverse and side effects [5,6] (Figure 1). Research in this area with more targeted approaches remains necessary for patients with high-risk neuroblastoma. We describe herein the synthesis and the evaluation of pyrido[2′,1′:2,3] imidazo[4,5-*c*]isoquinolin-5-amine derivatives as potential anticancer agents in the human neuroblastoma cell line.

**Scheme 1:**
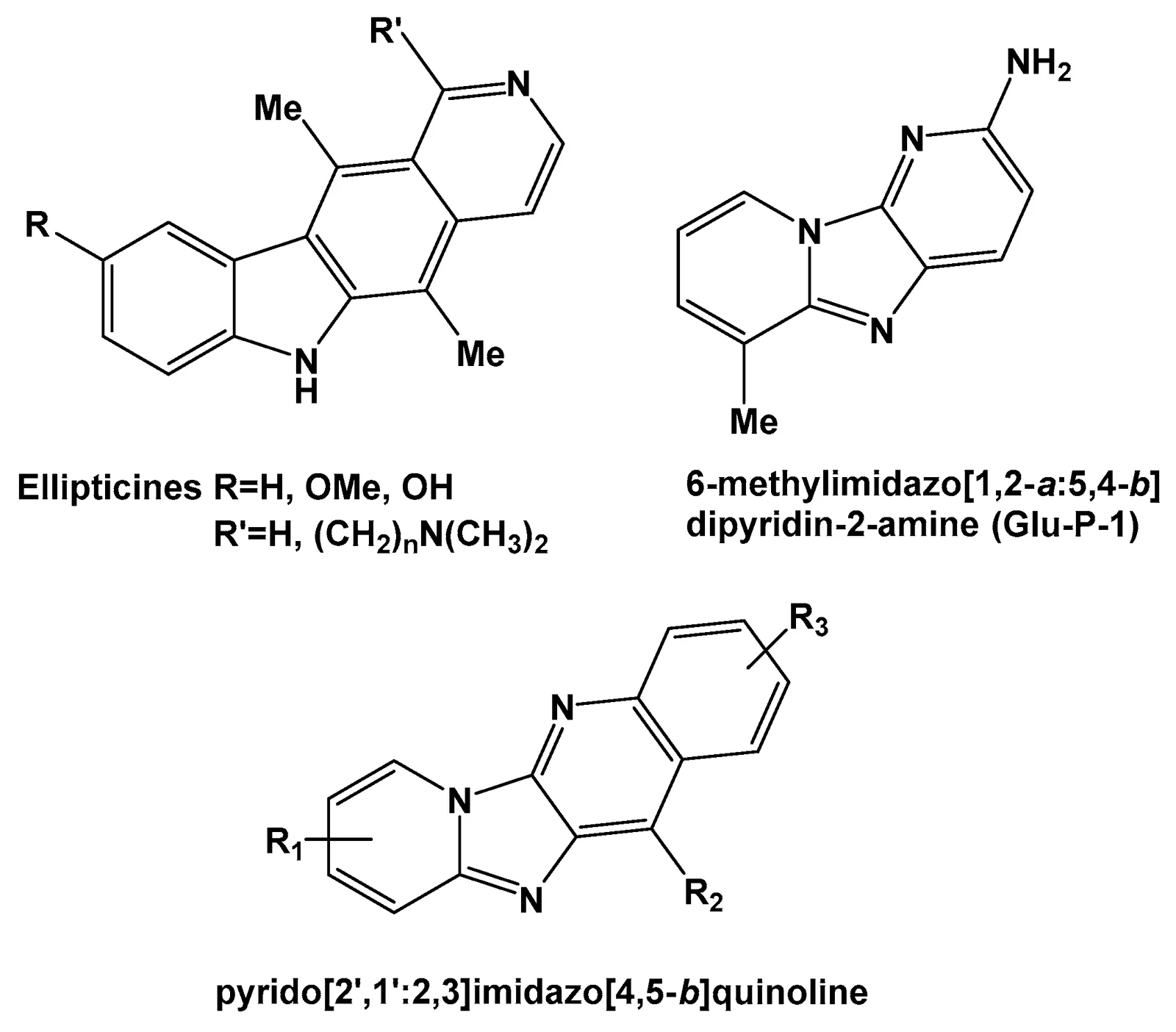
Ellipticines and analogues.

**Scheme 2:**
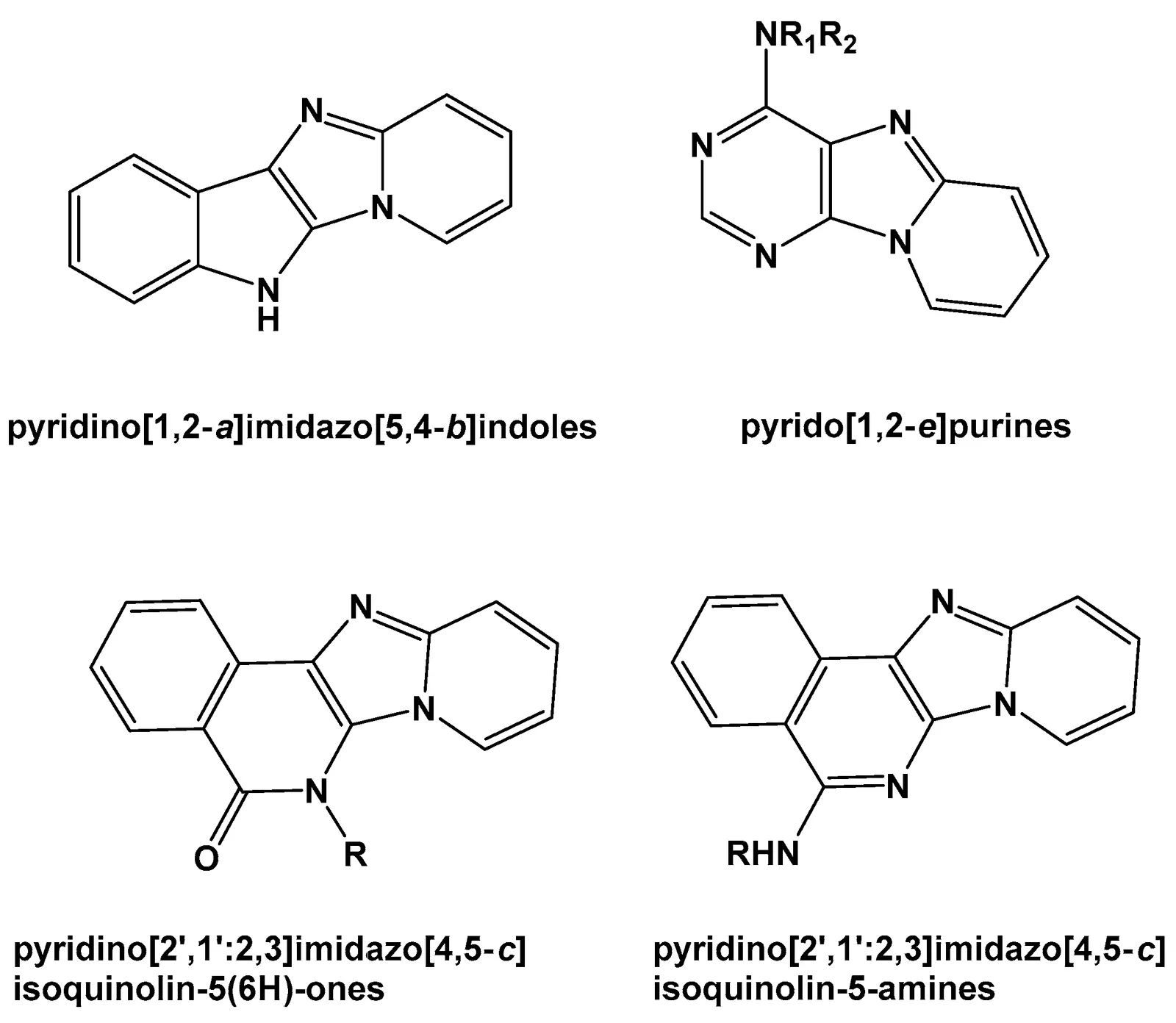
Example of fused imidazopyridines and pyrido [2′,1′:2,3]imidazo[4,5-*c*]isoquinolin-5-amines.

Carcinogenesis increases as long as apoptosis does not occur in many cancer cells [7,8]. It is well known that malignantly transformed cells play a crucial role in the progress of cancer. As a cell death mechanism, apoptosis is very important to avoid the proliferation of these cells. Therefore, many proteins that are involved in the apoptotic pathway are considered as important targets for several anticancer drugs. The imidazo[1,2-*a*]heterocyclic scaffold is a prominent structural core system present in numerous biologically active compounds and possesses a wide range of biological activities. Many investigations revealed the anticancer potential of imidazo[1,2-*a*]heterocyle derivatives [9,10,11,12,13,14]. They have also been introduced as anti-inflammatory [15,16] and anti-tumor agents [17,18] and are active in the treatment of central nervous diseases [19]. Moreover, it has been reported that several drugs with an imidazo[1,2-*a*]pyridine scaffold such as zolpidem or alpidem can be used to treat insomnia or anxiety disorders [20,21]. The Pyrido[2′,1′:2,3]imidazo[4,5-*c*]quinolines are isosteres of ellipticine known to have various anti-cancer activities [22,23,24,25,26]. After studying their synthesis by the Groebke–Blackburn–Bienaymé MCR [27,28] Scheme 1, we were interested via the same kind of reaction to the synthesis of pyrido [2′,1′:2,3]imidazo[4,5-*c*]isoquinolin-5-amines Scheme 2 [29]. We evaluated the anticancer potential of these structures in the human neuroblastoma cell line, the cytotoxicity was examined using the WST-1 test after 72 h of exposure to drugs.

**Figure 2:**
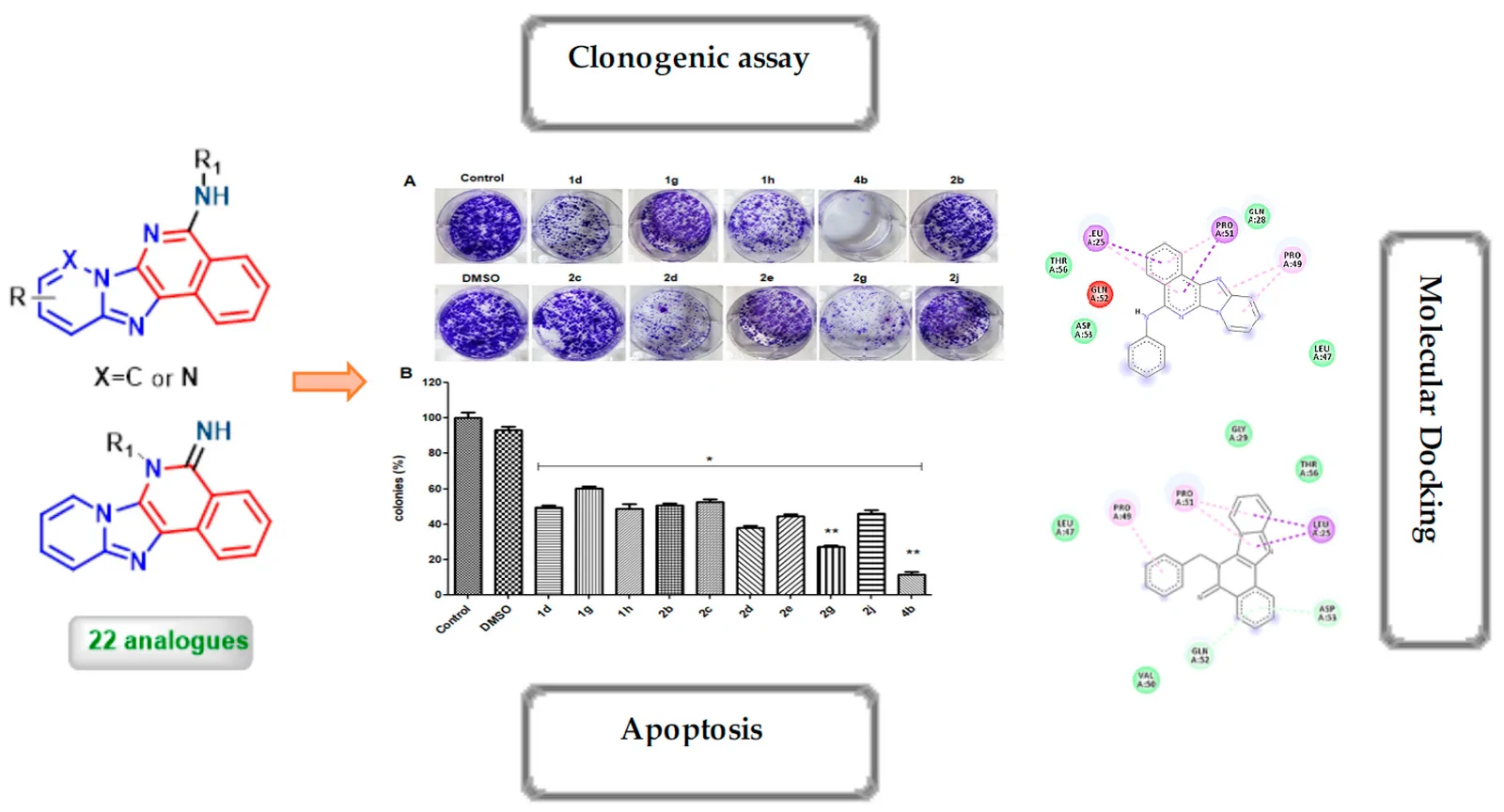
Scheme summarizing the results of this study. (**A**) Representative images of clonogenic assay in SH-SY5Y cells treated with the indicated drugs at 10 µM. (**B**) Percentage of viable clones after drug treatments with respect to untreated cells. * *p* < 0.01 significant difference from control group, ** *p* < 0.01 significant difference from other compounds.

This work describes the synthesis, molecular docking results and evaluation of the anticancer potential of pyrido[2′,1′:2,3]imidazo[4,5-*c*]isoquinolin-5-amine derivatives in human neuroblastoma in a preclinical model. Furthermore, molecular modelling investigations were performed to suggest possible binding modes of these inhibitors inside the active site of the target protein (Figure 2).

## 2. Results and Discussion

### 2.1. Chemistry

**Scheme 3:**
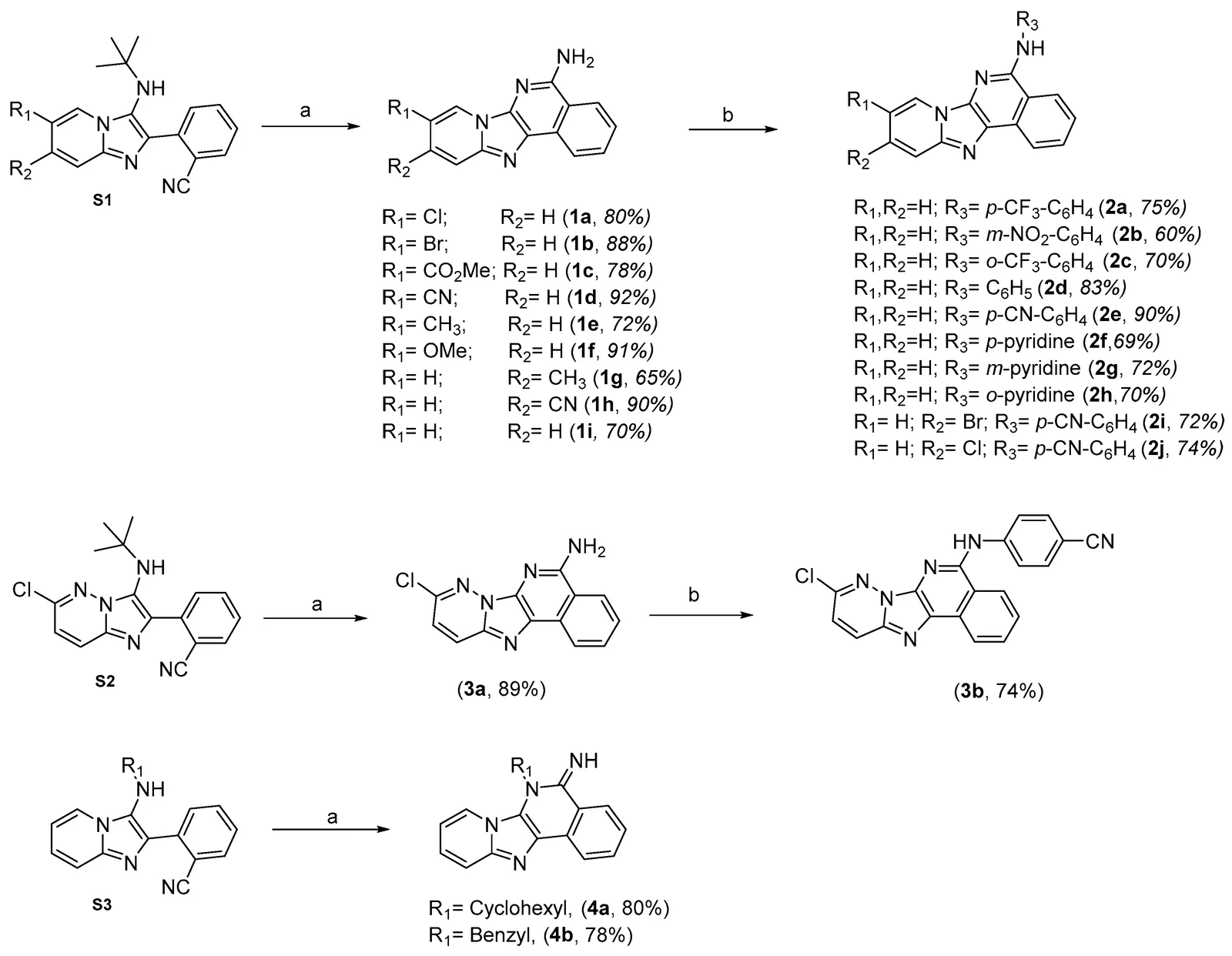
Synthetic route for various substituted pyridazino/pyrido[2′,1′:2,3]imidazo[4,5-*c*]isoquinolin-5-amines. Conditions: (a) TFA/DCM(1:1), rt; (b) R_2_-H, (hetero)aryl halide, Pd(OAc)_2_, Xantphos, Cs_2_CO_3_, PhMe, 120 °C.

The general synthetic route for the target compounds is outlined in Scheme 3. Compounds **1a**–**1i** were obtained from various products S1 that were obtained by a one-pot Groebke–Blackburn–Bienaymé MCR reaction according to our already published procedure [29] using 2-aminoheteroaryl, 2-formylbenzonitrile and *tert*-butylisocyanide in DCM at room temperature. During the N-deprotection step with a 1/1 mixture of DCM/TFA at room temperature, the electrophilic carbon of the cycano group underwent amine attack and cyclisation took place, leading to the pyrido[2′,1′:2,3]imidazo[4,5-*c*]isoquinolin-5-amines. To obtain the different substituted pyrido[2′,1′:2,3]imidazo[4,5-*c*]isoquinolin-5-amines **2a–2j** and **3a**, the synthetic strategy of the Buchwald reaction [30,31] was applied. Various aryl halides substituted in *para*, *meta* and *ortho* position were successfully introduced in good to excellent yields. Replacing *tert*-butyl by cyclohexyl and benzyl allowed us to synthesise novel imidazo[4,5-*c*]isoquinoline derivatives (**4a**, **4b**) with good yields via a one-step N-deprotection/cyclisation reaction.

### 2.2. Biological Studies

#### 2.2.1. Cell Viability Studies

The cytotoxic potential of compounds was firstly evaluated in human neuroblastoma cell lines in terms of decrease in cell viability. WST-1 methods were used to evaluate the cytotoxicity of the synthesized compounds. The changes in cell viability after 72 h drug exposure are shown in Table 1. It was found that all the compounds tested, except **1d**, **1h**, **2a**, **2f**, **2i**, **2j** and **3b**, showed moderate antiproliferative activity against the cancer cells. In particular, the IC50 values of compounds **1g**, **2d** and **4b** were found to be less than 10 µM.

**Figure 3:**
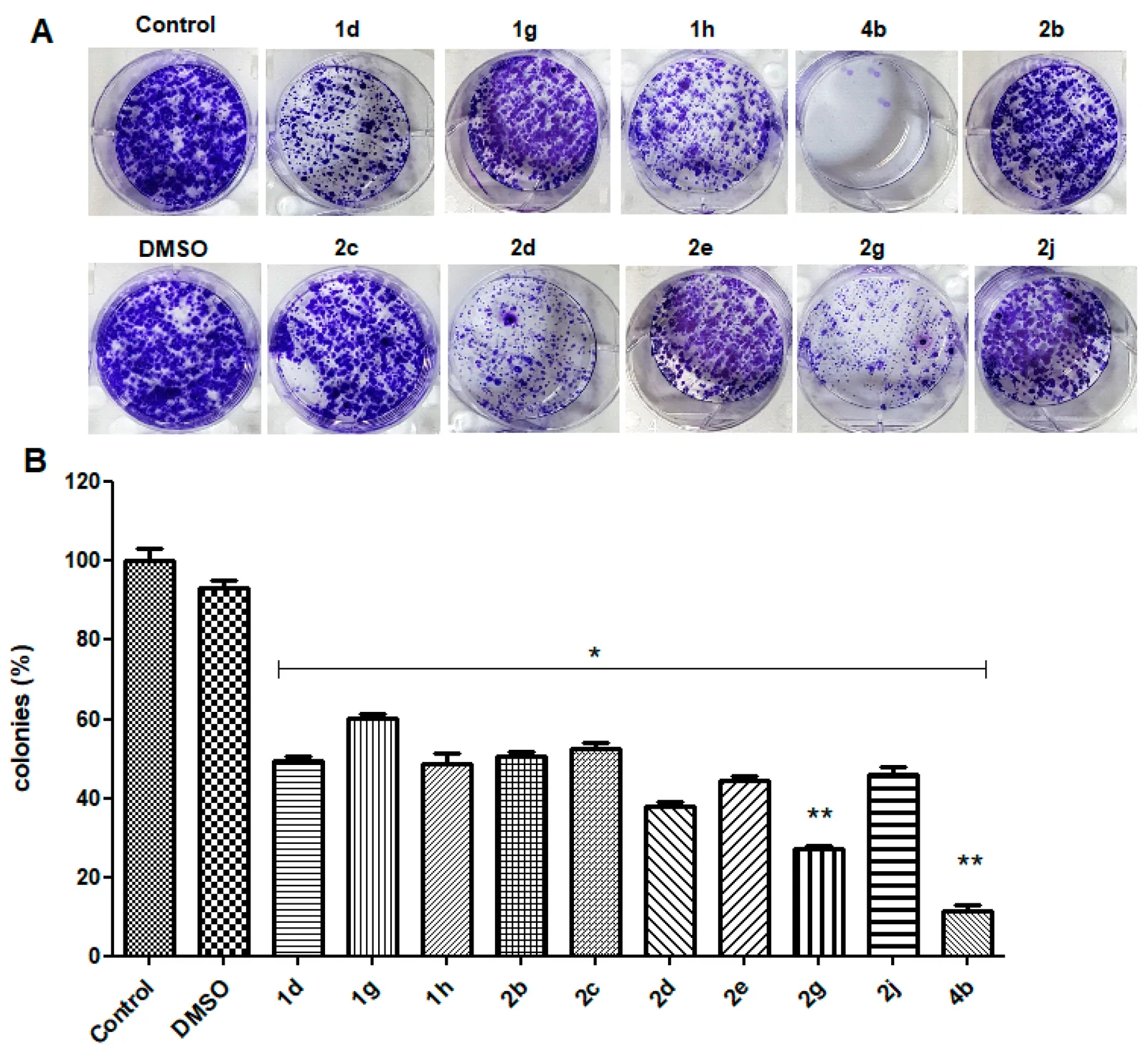
(**A**) Representative images of clonogenic assay in SH-SY5Y cells treated with the indicated drugs at 10 µM. (**B**) Percentage of viable clones after drug treatments with respect to untreated cells. * *p* < 0.01 significant difference from control group, ** *p* < 0.01 significant difference from other compounds.

The ability of cells to form colonies is crucial. The number of colonies was therefore counted 7 days after exposure to 10 µM of drugs that were selected after the cell viability analyses (Figure 3). We found that exposure to compounds **2d**, **2g** and **4b** significantly diminished the number of clones per field as compared to cells from the control group (*p* < 0.01), indicating the importance of further preclinical investigations.

#### 2.2.2. Protein Analysis

Based on the results obtained by the clonogenic assay, further analyses were conducted for compounds **2d**, **2g** and **4b** at three different concentrations (1–100 µM) in order to understand their possible roles in regulating protein levels including cleaved caspase 3, cleaved PARP, Bax and Bcl2, which are responsible for cell death. Results from the Western blot analysis showed that the treatment of SH-SY5Y cells with different concentrations of the selected compounds for 72 h upregulated some apoptotic proteins such as Bax, cleaved caspase-3 and cleaved PARP-1.

**Figure 4:**
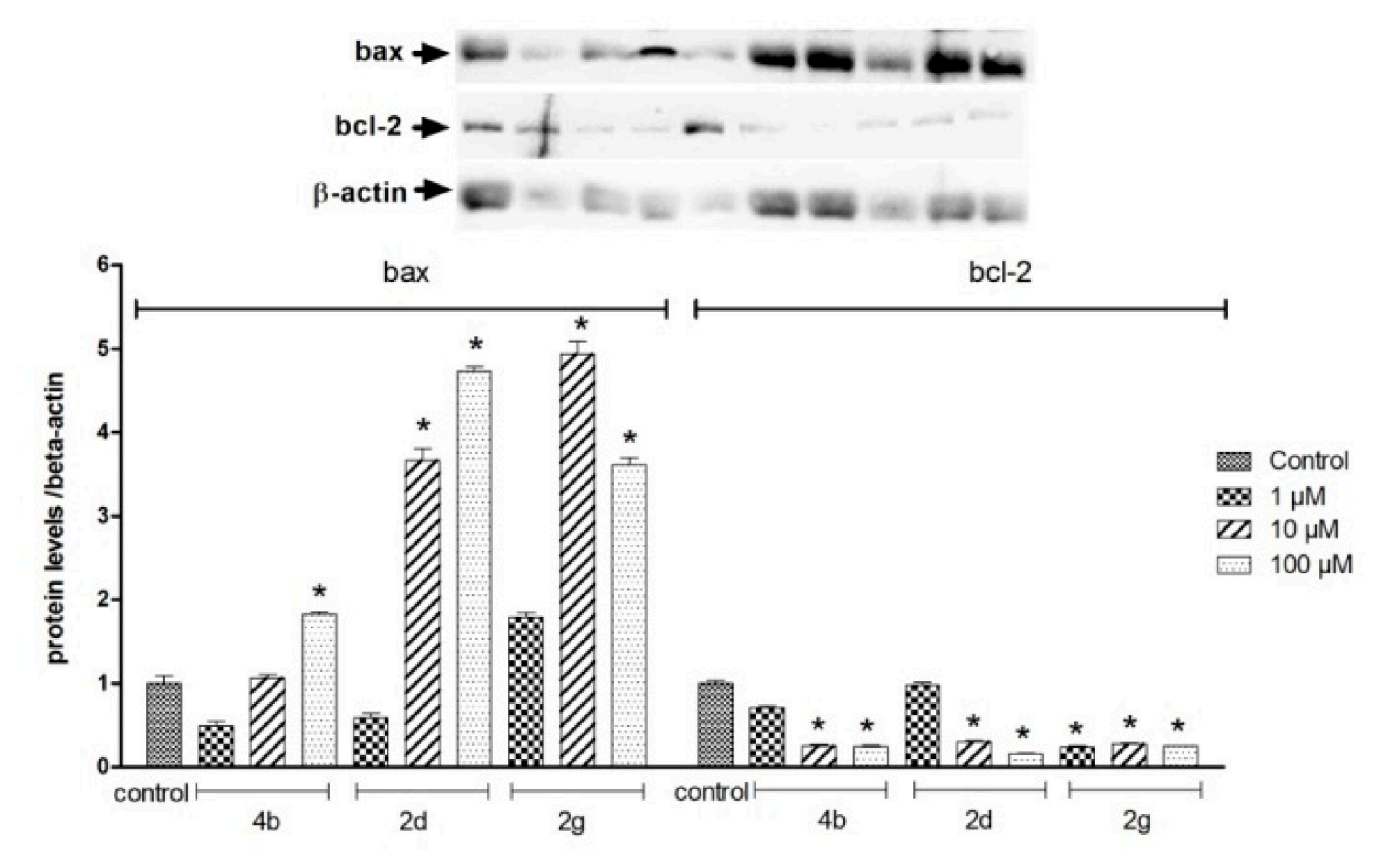
Changes in pro-apoptotic Bax and anti-apoptotic bcl-2 protein levels. Representative Western blots showing protein expressions of Bax and bcl-2 following the treatment of compounds **2d**, **2g** and **4b** at three concentrations (1, 10 and 100 µM). Graphs indicate the relative densitometric values of the proteins. Quantification of protein product was performed by densitometric scanning. Data were normalized by using the β-actin signal and are expressed as arbitrary densitometric units. Values are means SD; *n* = 5 in each group. * *p* < 0.01 significant difference from control group.

The balance between Bax and bcl-2 controls cell apoptosis. As shown in Figure 4, increases in Bax protein levels in cells treated at higher concentrations were accompanied by concomitant decreases of Bcl-2 protein expression when compared to the control group (Figure 4, *p* < 0.05), suggesting that the selected compounds have a regulatory potential for apoptosis by controlling the induction of mitochondrial dysfunction.

**Figure 5:**
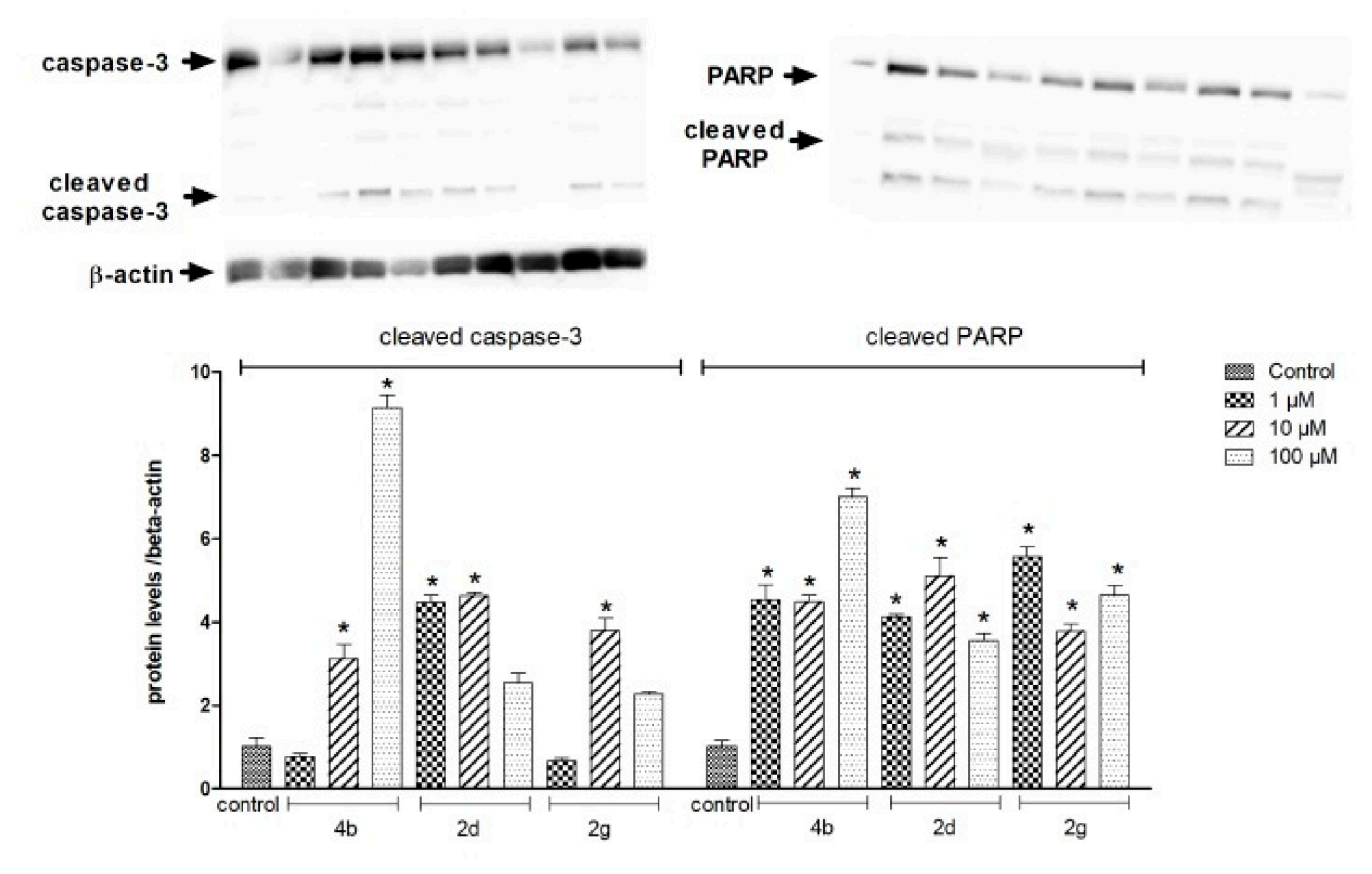
Changes in cleaved caspase-3 and cleaved PARP protein levels. Representative Western blots showing protein expressions of full-length or cleaved caspase-3 and PARP following treatments of compounds **2d**, **2g** and **4b** at three concentrations (1, 10 and 100 µM). Graphs indicate the relative densitometric values of the proteins. Quantification of protein product was performed by densitometric scanning. Data were normalized by using the β-actin signal and are expressed as arbitrary densitometric units. Values are means SD; *n* = 5 in each group. * *p* < 0.01 significant difference from control group.

The activation of effector caspases, particularly caspase 3, plays an important role in the actual execution of apoptosis [32,33]. In our study, the results showed that these compounds have the potential to regulate activation of the caspase 3 enzyme in a dose-dependent manner. The exposure of SH-SY5Y cells to compounds (**2d**, **2g** and **4b**), at 10 µM enhanced caspase-3 cleavage by 3.12, 4.62 and 3.79 fold, respectively (Figure 5) compared to the control group, thereby initiating the intrinsic apoptotic cascade.

One of several known cellular substrates of caspases is PARP-1. It is known that PARP-1 is involved in the prevention of DNA degradation and resistance to apoptosis. In recent studies, increased PARP-1 expression was shown to be associated with higher neuroblastoma stage and poor overall survival [34]. Thus, PARP-1 inhibitors are evaluated clinically for the treatment of brain tumors. However, the cleavage of PARP-1 by caspases is considered to be a hallmark of apoptosis [35] and caspase 3 is primarily responsible for the cleavage of this enzyme during programmed cell death [36]. In our study, the treatment of SH-SY5Y cells with compounds **2d**, **2g** and **4b** at lower concentrations also led to the cleavage of PARP-1 when compared to the control group (Figure 5). Our data clearly indicated that the compounds activated the intrinsic caspase cascade leading to caspase-3 activation that resulted in PARP-1 cleavage in the neuroblastoma cell line. With these Western Blot analyses, we addressed the question of whether the compounds in question can induce apoptotic cell death.

Despite recent improvements in patient risk stratification and genetic profiling, neuroblastoma remains the most lethal extracranial solid tumor in children. The combination of chemotherapeutic drugs including cisplatin, doxorubicin, cyclophosphamide and vincristine results in therapeutic failure because of chemoresistance [37]. Therefore, any alternatives with therapeutic potential should be studied in detail. In the present study, the in vitro proapoptotic and antiproliferative activities of these selected compounds are promising for further structural modifications and may provide a useful template for the design of new antitumoral agents.

### 2.3. Molecular Modelling Studies

#### 2.3.1. Protein and Ligand Preparation

For docking studies, the 3D structure of the pro-apoptotic Bax protein was retrieved from the RCSB protein data bank using its PDB id: 1F16 [38]. This protein structure, which consists of 192 residues with 9α helices without β sheets, was determined using the NMR solution technique. The 3D structure used was prepared by adding hydrogens, computing Gastiger charges, merging non-polar hydrogens and deleting water molecules. The preparation steps were carried out in the Bax protein by using the AutoDock Tools program (ADT) [39]. Further, the active site was selected to be around the α1 and α2 helices as being the most favorable pocket for the docking simulation.

#### 2.3.2. Molecular Docking Analysis

Molecular docking simulation was carried out with the pro-apoptotic Bax protein (1F16) and all ligands (i.e., pyrido-imidazole derivatives) using the AutoDock Vina software [40]. This tool functions using a Lamarckian Genetic Algorithm (LGA). The active site was input and the grid parameter file for the Bax protein was generated by fixing the number of grid points on the x = 4.528, y = −6.556, and z = −3.667 axes. AutoDock grids were calculated for regularly spaced points at intervals of 1.000 (Å) with dimensions of 30 × 28 × 22 (Å) and contained within a cube based on the helices α1 and α2 surrounding residues. The population size was set to 150. The maximum number of energy evaluations and generations was set to 500.000 and 1000, respectively. The rest of the docking parameters were set to the AutoDock Vina program default values. The LGA algorithm was used to find the best conformations in eight independent trials for each ligand.

To better understand whether or not the studied compounds had an effect on the activation of the Bax protein, molecular docking simulations of the three ligands (i.e., **2d**, **2g** and **4b**) with the α1 and α2 helices of the Bax trigger site were carried out. The simulations revealed that the ligands exhibit different and important types of interaction with significant docking scores (i.e., binding affinities). The interaction details of top three compounds with the active site residues are shown in Table 2.

**Figure 6:**
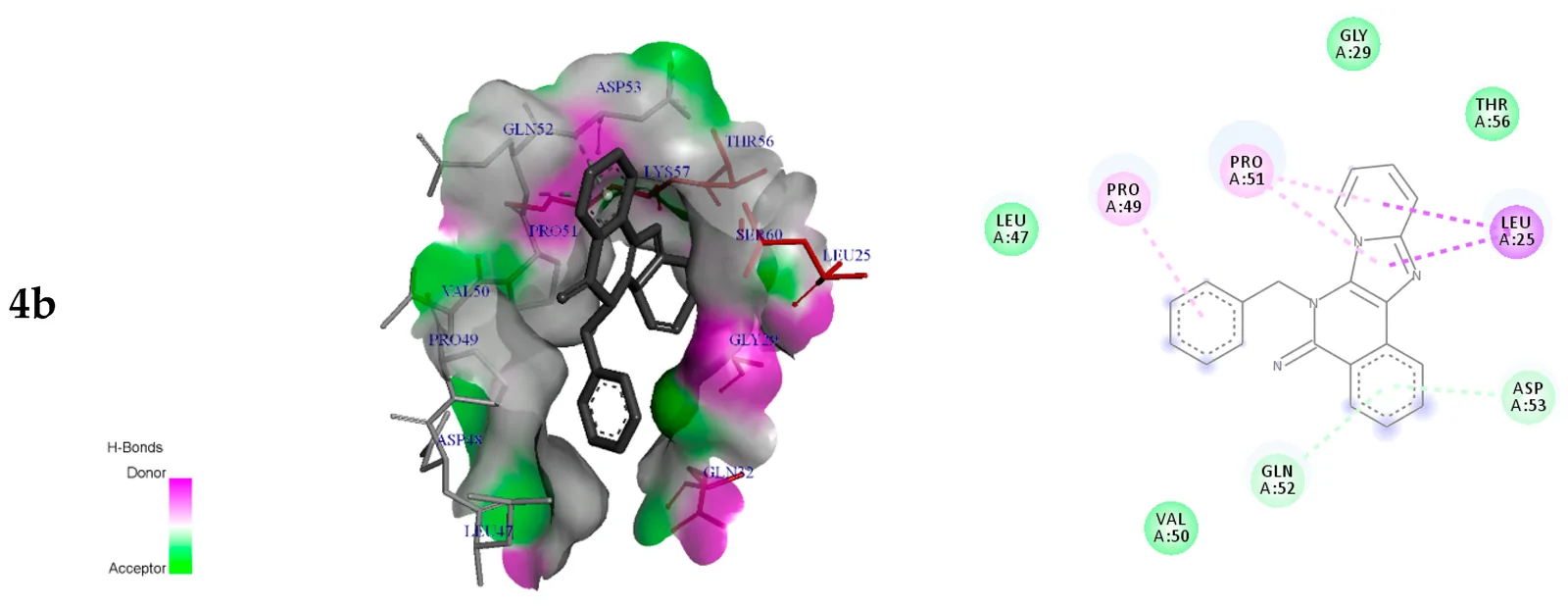

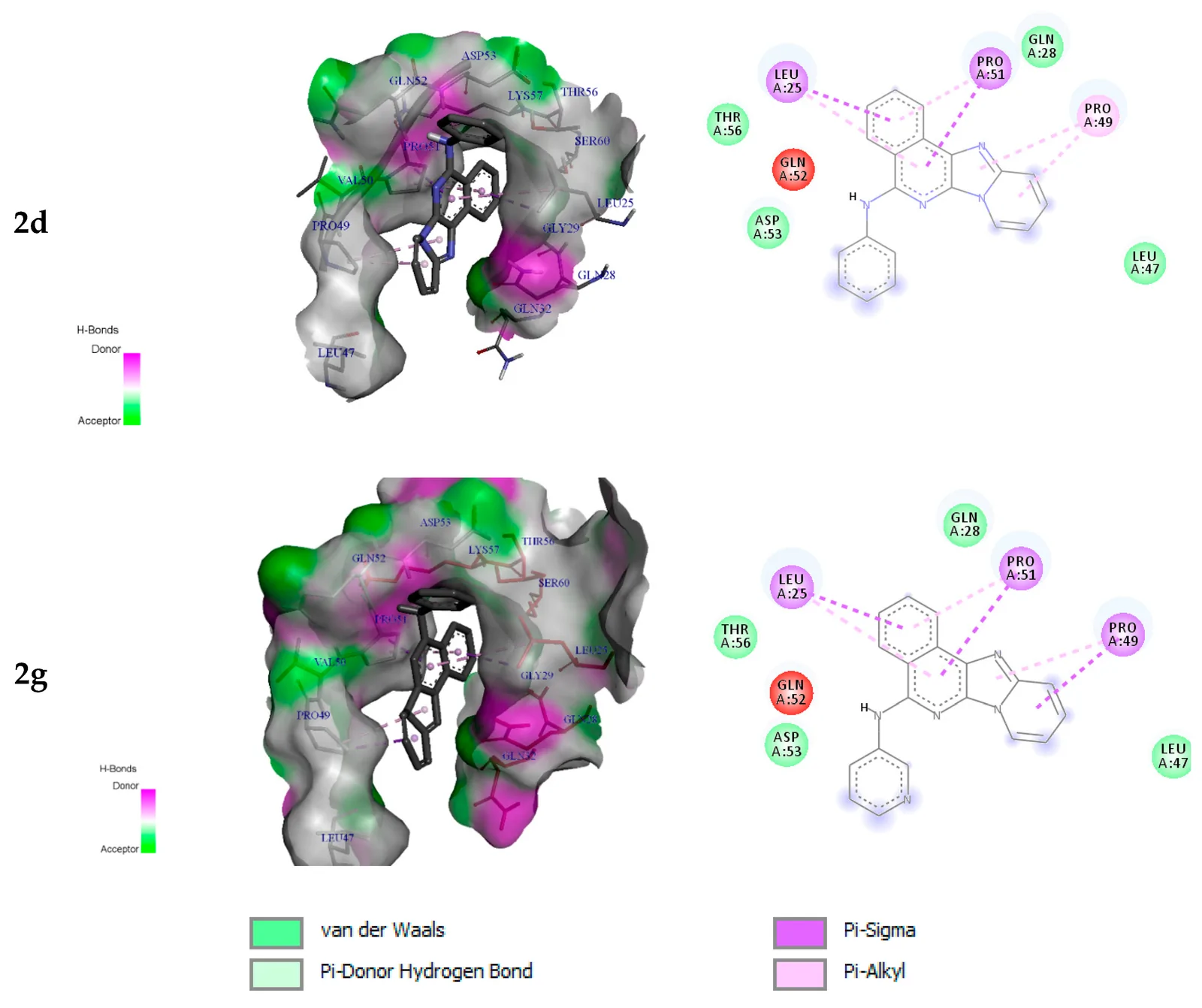
Hydrogen bond regions as exhibited by the active site (**left**). VDW and mixed/π-alkyl hydrophobic interactions (**right**) as exhibited by compounds **2d**, **2g** and **4b**.

On the one hand, non-classical hydrogen bond (HB) and mixed/π-alkyl hydrophobic such as π-sigma, π-alkyl and π-donor H-bond interactions were found to have major effects in initiating the activation of the Bax protein. The examination of these types of interactions is illustrated in Figure 6. In this figure, green and pink regions indicate the areas where HB-acceptor and HB-donor areas are preferred, respectively. The main interacting amino acids were Leu25, Pro49 and Pro51 for the three docked ligands.

**Figure 7:**
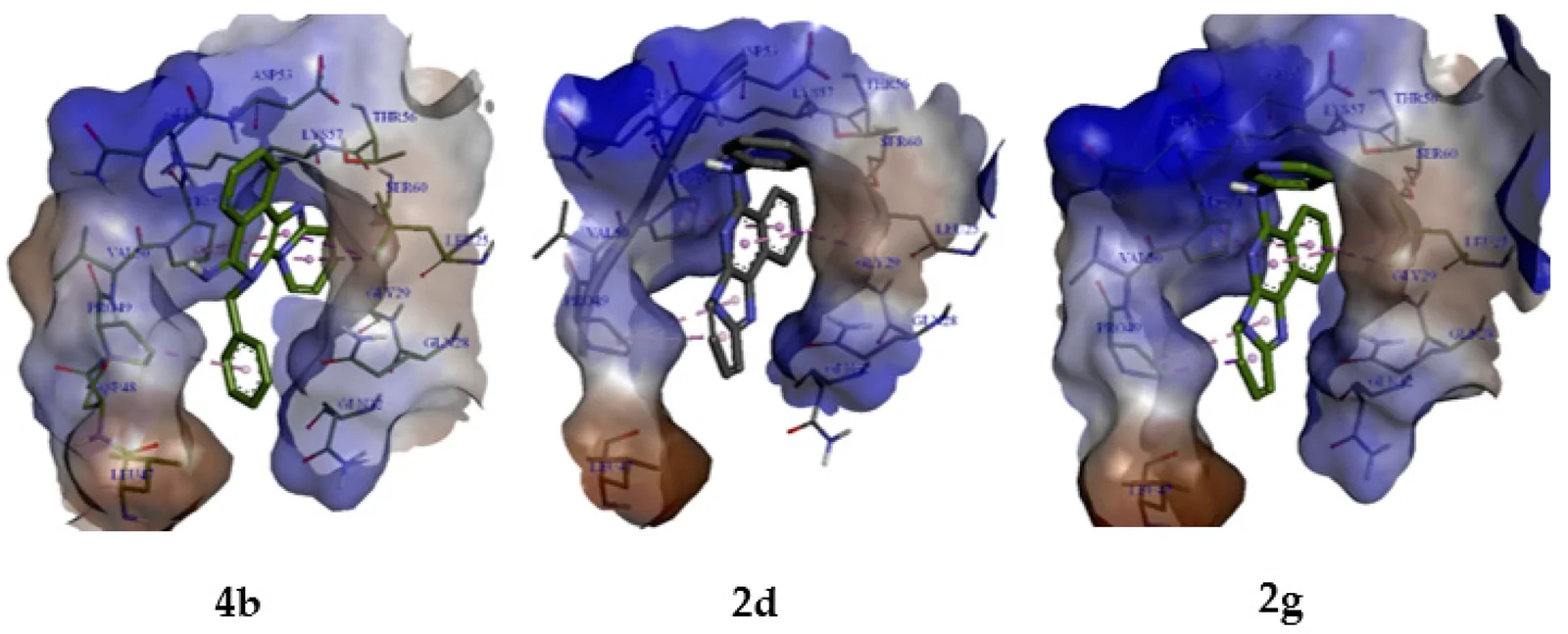
Hydrophobic area as exhibited by the active site along with compounds **2d**, **2g** and **4b**.

Additionally, both hydrophobic and hydrophilic amino acid residues of the active site groove played a contributing role in the interaction with the most active ligands (Figure 7). Indeed, the observed types of interactions of the selected compounds with the hydrophobic matrix of the active site could help in explaining the increase in the antiproliferative effect of these types of cells.

**Figure 8:**
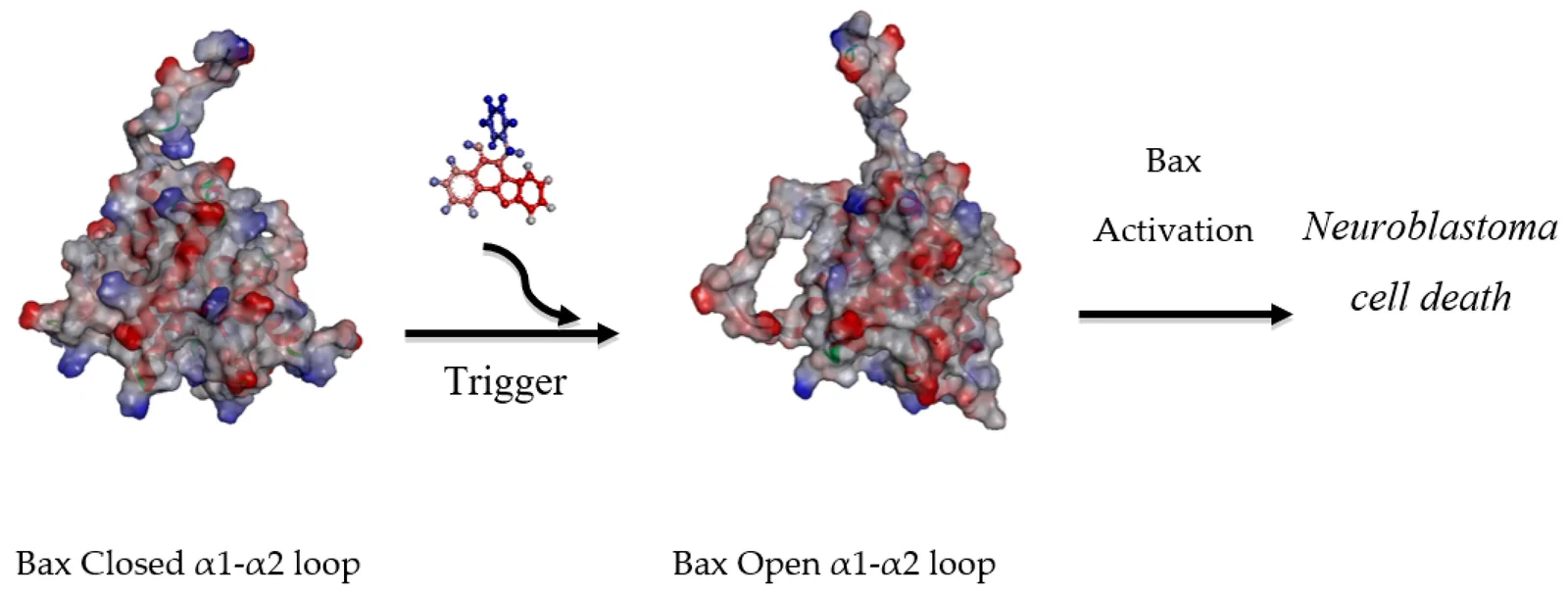
Predicted model of Bax activation initiated by displacement of the α1-α2 loop from a closed to an open conformation induced by the ligands examined in the present study.

All in all, it can be concluded that the initiation of Bax activation, which is taken away by the α1-α2 loop displacement, is due to exposure of the 12th to the 24th amino acid residues (Figure 8). As can be seen, the predicted model of Bax activation through displacement of the α1-α2 loop from a closed to an open conformation was induced by the selected compounds.

## 3. Materials and Methods

### 3.1. Chemistry Methods

#### 3.1.1. General Information

The reactions were monitored by thin-layer chromatography (TLC) analysis using silica gel (60 F254) plates. Compounds were visualized by UV irradiation. Flash column chromatography was performed on silica gel 60 (230–400.13 mesh, 0.040–0.063 mm). Melting points (mp (°C)) were taken on samples in open capillary tubes and are uncorrected. The infrared spectra of compounds were recorded on a Thermo Scientific Nicolet iS10 and are given in cm^−1^. ^1^H and ^13^C NMR spectra were recorded on a Bruker DPX 250 MHz (^13^C, 62.9 MHz), Bruker advance II 250.13 MHz (^13^C, 63 MHz), Bruker advance 400.13 MHz (^13^C, 101 MHz), or on a Bruker advance III HD nanobay 400.13 MHz (^13^C, 101 MHz). Chemical shifts are given in parts per million from tetramethylsilane (TMS) as internal standard. Coupling constants (J) are reported in hertz (Hz). High-resolution mass spectra (HRMS) were performed on a Maxis Bruker 4G. The information is contained within Supplementary Material.

#### 3.1.2. General Groebke–Blackburn–Bienaymé MCR Procedure for the Synthesis of (**S1**, **S2** and **S3**)

To a solution of amine (1.06 mmol, 1 equiv) in 1 mL dichloromethane, 2- cyanobenzaldehyde (1.16 mmol, 1.1 equiv) and perchloric acid (2–3 drops, 70% in water) were added and left at room temperature for 1 h, then isonitrile (1.16 mmol, 1.1 equiv) was introduced. After completion of the reaction (controlled by TLC), the mixture was concentrated under reduced pressure and the crude material was purified by flash chromatography on silica gel to provide the expected product.

#### 3.1.3. General *N*-Deprotection-cyclization Procedure

Compounds **1a**–**1j**, **3a** and **4a**, **4b** were prepared by following the *N*-deprotection-cyclization procedure, to a solution of **S1**, **S2** or **S3** (0.49 mmol, 1 equiv.) in dichloromethane (5 mL), TFA (5mL) was added. After completion of the reaction (controlled by TLC), the mixture was concentrated and the residue was dissolved in water. The pH solution was adjusted at 8 and the resulting precipitate was collected by filtration, washed with water and dried. The crude product was purified by crystallization with Et_2_O.

#### 3.1.4. Buchwald–Hartwig Cross-Coupling Procedure

Compounds **2a**–**2j** and **3b** were prepared by following the Buchwald–Hartwig cross-coupling procedure [29]. Under argon atmosphere, Pd(OAc)_2_ (2 mg, 0.0085 mmol, 0.02 equiv.), Xantphos (4,5-bis(diphenylphosphino)-9,9-dimethylxanthene) (5 mg, 0.0085 mmol, 0.02 equiv.) were introduced in a round-bottomed flask and diluted in dry toluene (1 mL) and left at room temperature for 1 h. Meanwhile, in a two-necked round-bottomed flask, aryl halide (0.426 mmol, 1.00 equiv.), 1(**a**–**i**) or 3a (100 mg, 0.426 mmol, 1.00 equiv.) and Cs_2_CO_3_ (558 mg, 1.70 mmol, 4.00 equiv.) were introduced in dry toluene (5 mL). To this suspension, the previously pre-formed Pd-catalyst was added. The resulting mixture was flushed with argon and heated at 120 °C. After completion of the reaction (controlled by TLC), toluene was removed under reduced pressure, water (10 mL) was added and the resulting aqueous phase was extracted with ethyl acetate. The combined organic layer was dried over magnesium sulfate and concentrated under reduced pressure. The crude product was purified by flash chromatography on silica gel.

### 3.2. Biological Methods

#### 3.2.1. Cell Culture and Treatments

The human neuroblastoma cell line (SH-SY5Y) used in this study was purchased from the American Type Culture Collection (ATCC, Catalog #CRL-2266, Manassas, VA, USA). Cells were suspended in complete Dulbecco’s modified Eagle Medium (DMEM) (Life Technologies, Gibco BRL, Grand Island, NY, USA) supplemented with 10% fetal bovine serum (FBS, Hyclone, ATCC, Manassas, VA, USA), 1% penicillin and streptomycin (100 U/mL, Invitrogen, Fisher Scientific, Illkirch, France) and plated in cell culture dishes. The cultures were maintained at 37 °C in 5% CO_2_ 95% humidified atmosphere. After reaching 85% confluence, cells were transferred to 96-well plates or culture dishes. For WST-1 experiments, cells were seeded into 96-well cell culture plates at a density of 2 × 10^3^ cells per well and allowed to adhere for 24 h. The culture medium was then replaced with fresh medium containing compounds (100, 10, 1, 0.1 µM) and incubated in a 5% CO_2_ incubator.

For protein analysis, cells were seeded into 6-well cell culture plates at a density of 0.5 × 10^6^ cells/well and allowed to adhere to the surface for 24 h. The stock solutions of the test compounds (5 mM) were prepared in sterile DMSO and these stocks were then appropriately diluted with the complete culture medium and DMSO levels were maintained below 1% in the test concentrations.

#### 3.2.2. Cell Proliferation Assay

SH-SY5Y cells were seeded at a density of 2 × 10^3^ cells/well into 96-well plates and incubated for 24 h. After 24 h, the cells were treated with the synthesized compounds at 0.1, 1, 10 and 100 µM concentrations for 72 h. Following the 72 h exposure, the cell viability was determined using the WST-1 assay. Cell viability was evaluated using Cell Proliferation Reagent WST-1 (Roche, France) according to the manufacturer’s instructions. The absorbance was measured at 440 nm and 600 nm using a microplate reader (VersaMax, Molecular Devices, USA). Percent survival was plotted relative to vehicle control cells, which were normalized to 100% survival. Culture medium with WST-1 without cells was used for background control.

#### 3.2.3. Clonogenic Survival Assay

This assay is an in vitro cell survival and proliferation assay based on the ability of a single cell to grow into a colony [41,42,43]. Briefly, 1500 cells were mixed gently in 2 mL media and plated on a 6-well plate. After being incubated for 24 h, the drugs were applied. The media which contain drugs were refreshed weekly and cells were grown for 2 to 3 weeks. At the end, the cells were washed with phosphate-buffered saline and stained with 10% crystal violet. Colonies with a diameter of more than 50 cells were counted. The experiment was repeated three times.

#### 3.2.4. Protein Analysis

SH-SY5Y cells (0.5 × 10^6^ cell/well) were seeded into 6-well plates and incubated for 24 h. After 24 h, the cells were treated with the synthesized compounds at three different concentrations, 1, 10 and 100 µM for 72 h. Following treatment, the cells were washed with ice-cold 1X PBS and lysed in 1X cell lysis buffer (phosphatase and protease inhibitors added). These lysates were quantified using the BCA protein assay. Then, Western blot assays were done by using cell lysates.

#### 3.2.5. Western Blotting

Western blotting was performed by loading 30 μg protein on 10% (*w/v*) tris-glycine denaturing gels and separating the proteins by electrophoresis, then transferring them to a PVDF membrane. After blocking, the membrane was incubated with primary antibodies, anti-PARP rabbit monoclonal antibody (1:1000, Cell Signaling Technology, Invitrogen Fisher Scientific, Illkirch, France), anti-caspase-3 rabbit monoclonal antibody (1:1000, Cell Signaling Technology), anti-Bax rabbit monoclonal antibody (1:1000, Cell Signaling Technology), anti-Bcl-2 rabbit monoclonal antibody (1:1000, Cell Signaling Technology) at +4 °C overnight. Following incubation, the membrane was incubated with anti-β-actin mouse monoclonal antibody (1:1000, Cell Signaling Technology) at room temperature for 1 h. After washing, the membrane was incubated with infrared labeled secondary antibodies (Odyssey Western Blotting kit, LI-COR Biosciences, Lincoln, NE, USA) at room temperature for 1h. Proteins were visualized, and quantified by scanning the membrane on an Odyssey Infrared Imaging System (LI-COR Biosciences, Lincoln, NE, USA) with both 700- and 800-nm channels.

#### 3.2.6. Statistical Analysis

Data were expressed as means ± standard deviation (SD) for five or three independent experiments in triplicate. Comparisons of means between groups were performed by one-way analysis of variance (ANOVA) followed by Tukey’s post hoc test. *p* < 0.05 was considered statistically significant.

### 3.3. Computational Study

The 1F16 (Closed bax protein) [38] solution structure of a Pro-apoptotic Bax protein was prepared according to the protein preparation module in the AutoDockTools (ADT) package. Rigid and flexible conformations were prepared for all amino acids. Three-dimensional (3D) ligand structures were drawn and minimized using the Gaussview 05 and Gaussian programs [44], respectively. A semi-flexible docking study was performed with the AutoDock Vina program [40]. The best docking conformations of the compounds with the highest activities (i.e., **4b**, **2d** and **2g**) were selected to analyze different molecular interactions within the Bax protein active pocket using the Discovery studio program [45].

## 4. Conclusions

In summary, we report herein the design and synthesis of tetra-heterocycles functionalized by amine. The cytotoxic potential of these derivatives was evaluated in human neuroblastoma cell lines and showed moderate antiproliferative activity against the cancer cells with IC50 values below 10 µM for a few derivatives. The studies carried out have shown, on the one hand, that some compounds had a regulatory potential of apoptosis via control of the induction of mitochondrial dysfunction, and on the other hand, that these compounds had a potential to regulate the caspase 3 enzyme activation in a dose-dependent manner. These compounds enhance caspase-3 cleavage, thereby initiating the intrinsic apoptotic cascade. We show that the in vitro proapoptotic and antiproliferative activities of these selected compounds are promising for further structural modifications and may provide a useful template for the design of new antitumoral agents. Moreover, the molecular docking studies indicated that our synthetic derivatives have significant binding affinities with the target protein. As neuroblastoma is the most lethal extracranial solid tumor in children, any approaches with an interesting therapeutic potential should be studied in detail. The predicted model of Bax activation through displacement of the α1-α2 loop from a closed to an open conformation (Figure 8) was induced by the selected compounds. Compounds **2d**, **2g** and **4b** possessed significant proapoptotic and antiproliferative activities and may be useful as lead molecules for designing new antitumoral agents.

## Figures and Tables

**Table 1:** Cell viability analysis of newly synthesized compounds in human neuroblastoma cell lines. All values are means ± SDs (*n* = 5).

Compound	IC_50_ (µM)	% Cell Viability ± S.D.			
		0.1 µM	1 µM	10 µM	100 µM
**1a**	30.92	79.26 ± 7.30	74.37 ± 3.07	56.05 ± 1.49	42.28 ± 3.54
**1b**	20.22	78.13 ± 7.12	82.00 ± 7.82	80.76 ± 17.03	2.82 ± 0.68
**1c**	63.57	73.13 ± 6.09	64.93 ± 8.65	59.36 ± 3.36	47.06 ± 2.76
**1d**	>100	67.87 ± 1.59	74.17 ± 1.80	68.67 ± 1.23	58.14 ± 5.16
**1e**	29.72	74.28 ± 7.46	76.26 ± 9.43	69.99 ± 5.20	32.25 ± 1.75
**1f**	62.75	95.60 ± 7.30	83.16 ± 2.45	61.48 ± 3.22	48.50 ± 1.59
**1g**	4.31	67.22 ± 2.46	77.39 ± 13.70	48.98 ± 2.88	2.8 ± 2.13
**1h**	>100	98.76 ± 18.97	88.84 ± 9.17	63.66 ± 3.69	77.20 ± 10.89
**2a**	>100	103.32 ± 7.22	124.52 ± 24.18	122.45 ± 3.75	53.93 ± 10.50
**2b**	75.51	89.66 ± 5.59	77.28 ± 6.50	58.54 ± 2.45	41.20 ± 6.35
**2c**	65.03	77.25 ± 0.46	73.04 ± 1.71	68.01 ± 6.44	42.65 ± 2.82
**2d**	9.68	79.99 ± 2.81	71.43 ± 1.99	69.37 ± 6.58	2.68 ± 0.78
**2e**	21.09	81.18 ± 2.57	76.57 ± 7.64	61.70 ± 3.10	32.39 ± 11.92
**2f**	>100	89.52 ± 10.33	83.36 ± 10.20	70.19 ± 4.08	52.03 ± 0.82
**2g**	20.54	96.81 ± 11.13	81.72 ± 2.09	59.98 ± 3.53	30.02 ± 3.13
**2h**	91.71	85.05 ± 8.52	77.79 ± 5.59	72.48 ± 4.91	45.71 ± 1.66
**2i**	>100	99.39 ± 5.10	82.60 ± 3.65	89.10 ± 8.44	85.14 ± 6.14
**2j**	>100	96.16 ± 17.53	82.43 ± 14.56	81.94 ± 8.51	109.05 ± 9.13
**3a**	43.51	98.72 ± 6.89	90.64 ± 6.24	68.55 ± 4.49	38.98 ± 2.26
**3b**	>100	79.29 ± 4.73	83.81 ± 2.62	85.39 ± 1.74	106.26 ± 10.35
**4a**	15.53	69.08 ± 5.18	72.08 ± 3.46	76.77 ± 8.51	17.89 ± 2.70
**4b**	1.68	73.29 ± 2.02	63.74 ± 5.20	27.16 ± 2.43	9.24 ± 2.60

**Table 2:** Most non bond interactions between Pro-apoptotic Bax protein and top 3 compounds.

Compound	Binding Free Energies (Kcal/mol)	Residues in Contact	Interaction Type	Distance in Å
**4b**	−6.9	Leu25 Pro49 Pro51 Gln52 Asp53	π-sigma π-alkyl π-alkyl π-donor H-bond π-donor H-bond	3.89 4.65 4.34 3.95 4.16
**2d**	−6.7	Leu25 Pro49 Pro51	π-sigma π-alkyl π-sigma	3.74 4.03 3.91
**2g**	−6.6	Leu25 Pro49 Pro51	π-sigma π-sigma π-sigma	3.74 3.56 3.91

## Data Availability

The chemical molecules studied in this article are described in reference [29] with the exception of products **4a** and **4b** which are described in Supplementary Material.

## Supplementary Materials

The following are available online at https://www.mdpi.com/article/10.3390/ph14080750/s1.
