# Correction: Tber et al. Pyrido[2′,1′:2,3]imidazo[4,5-*c*]isoquinolin-5-amines as Potential Cytotoxic Agents Against Human Neuroblastoma. *Pharmaceuticals* 2021, *14*, 750

**DOI:** 10.3390/ph19081297

**Published:** 2026-08-17

**Authors:** Zahira Tber, Mohammed Loubidi, Jabrane Jouha, Ismail Hdoufane, Mümin Alper Erdogan, Luciano Saso, Güliz Armagan, Sabine Berteina-Raboin

**Affiliations:** 1 Institut de Chimie Organique et Analytique ICOA, Université d’Orléans-Pôle de Chimie, UMR CNRS 7311, Rue de Chartres-BP 6759, CEDEX 2, 45067 Orléans, France; tber_zahira@yahoo.com (Z.T.); m.loubidi@gmail.com (M.L.); jabranejouha@gmail.com (J.J.); 2 Laboratory of Molecular Chemistry, Faculty of Sciences Semlalia, Cadi Ayyad University, Marrakech BP 2390, Morocco; i.hdoufane@gmail.com; 3 Department of Physiology, School of Medicine, Izmir Katip Çelebi University, Izmir 35620, Turkey; alpero86@gmail.com; 4 Department of Physiology and Pharmacology Vittorio Erspamer, Sapienza University of Rome, P. le Aldo Moro 5, 00185 Rome, Italy; luciano.saso@uniroma1.it; 5 Department of Biochemistry, Faculty of Pharmacy, Ege University, Bornova 35100, Turkey

**Error in Figures**

In the original publication [1], there was a mistake in Figures 4 and 5 as published. An error occurred during figure assembly due to incorrect labeling of blot image files. The error was limited to figure preparation and did not affect the underlying experimental data, data interpretation, or the conclusions of the study. The corrected Figure 4 and Figure 5 appear below. The authors state that the scientific conclusions are unaffected. This correction was approved by the Academic Editor. The original publication has also been updated.

## Figures and Tables

**Figure 4:**
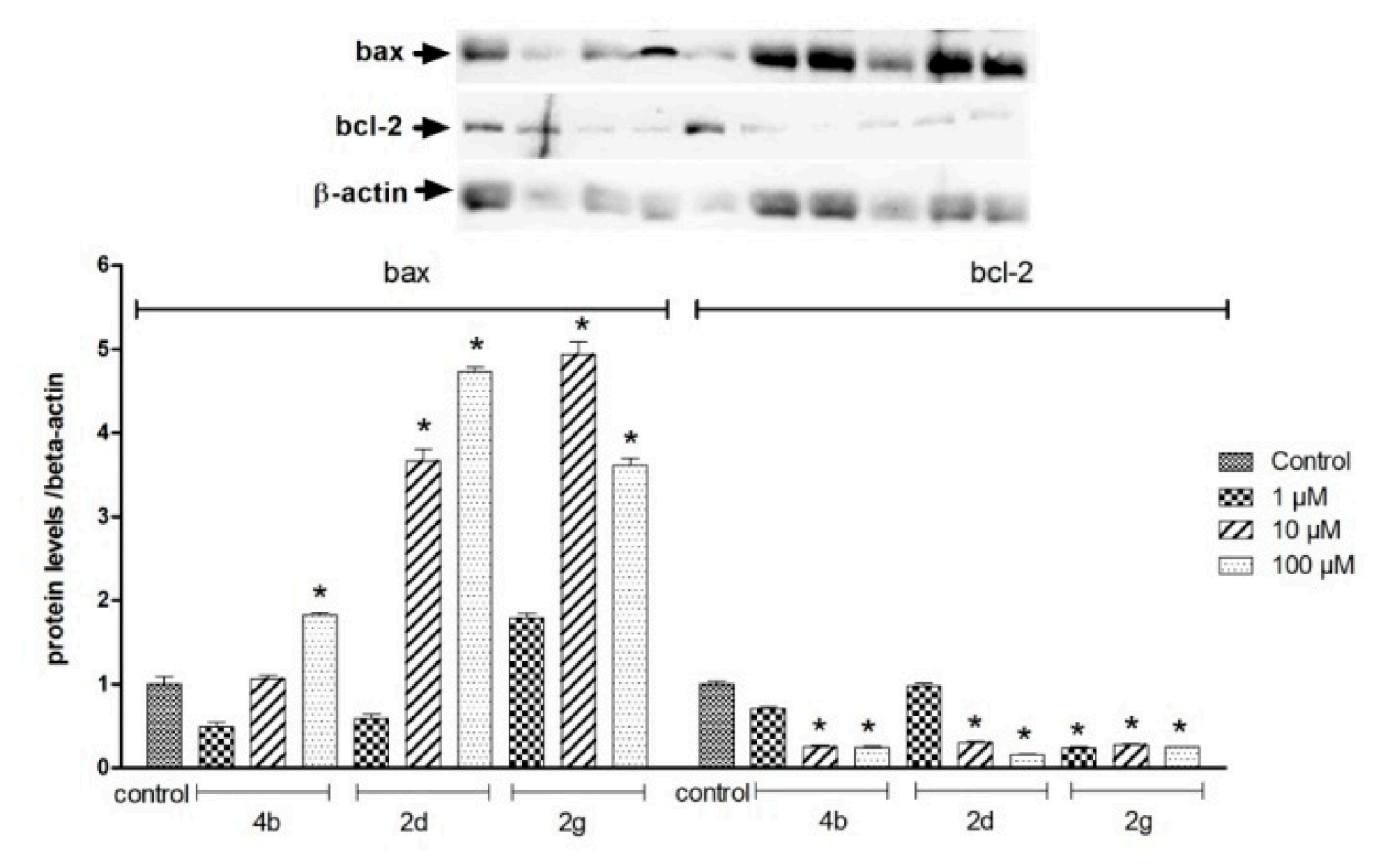
Changes in pro-apoptotic Bax and anti-apoptotic bcl-2 protein levels. Representative Western blots showing protein expressions of Bax and bcl-2 following the treatment of compounds **2d**, **2g** and **4b** at three concentrations (1, 10 and 100 µM). Graphs indicate the relative densitometric values of the proteins. Quantification of protein product was performed by densitometric scanning. Data were normalized by using the β-actin signal and are expressed as arbitrary densitometric units. Values are means SD; *n* = 5 in each group. * *p* < 0.01 significant difference from control group.

**Figure 5:**
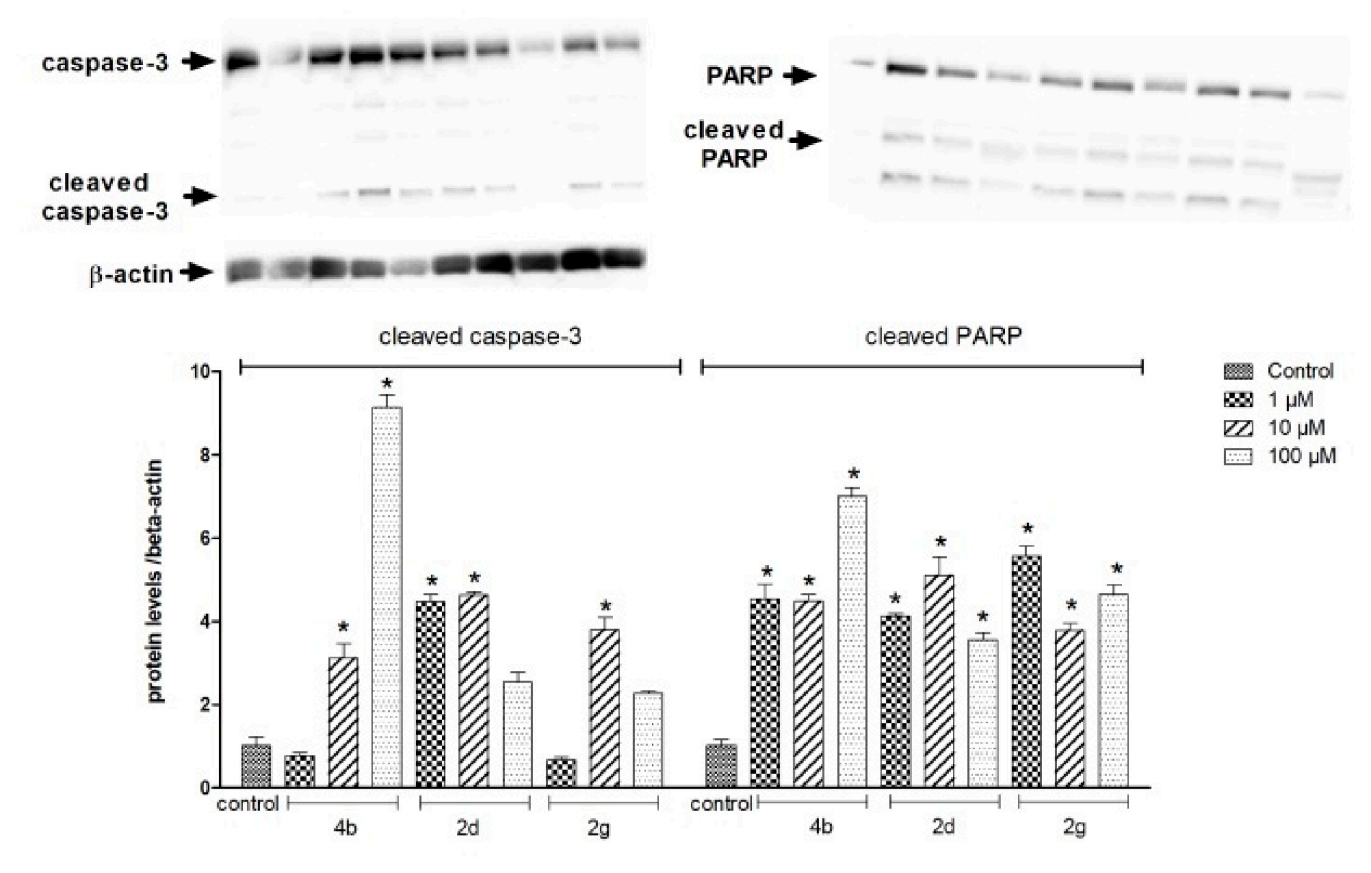
Changes in cleaved caspase-3 and cleaved PARP protein levels. Representative Western blots showing protein expressions of full-length or cleaved caspase-3 and PARP following treatments of compounds **2d**, **2g** and **4b** at three concentrations (1, 10 and 100 µM). Graphs indicate the relative densitometric values of the proteins. Quantification of protein product was performed by densitometric scanning. Data were normalized by using the β-actin signal and are expressed as arbitrary densitometric units. Values are means SD; *n* = 5 in each group. * *p* < 0.01 significant difference from control group.
