# Supplementary material for: Pyrido[2′,1′:2,3]imidazo[4,5-c]isoquinolin-5-amines as Potential Cytotoxic Agents against Human Neuroblastoma
Source: Pharmaceuticals (Basel). 2021 Jul 30;14(8):750. doi: 10.3390/ph14080750 (PMC8399734; doi:10.3390/ph14080750)

pharmaceuticals

MDPI

Article

Pyrido[2′,1′:2,3]imidazo[4,5-*c*]isoquinolin-5-amines as potential cytotoxic agents against human neuroblastoma

Zahira Tber [1], Mohammed Loubidi [1], Jabrane Jouha [1], Ismail Hdoufane [2], Mümin Alper Erdogan [3], Luciano Saso [4], Güliz Armagan [5,*] and Sabine Berteina-Raboin [1,*]

[1] Institut de Chimie Organique et Analytique ICOA, Université d'Orléans-Pôle de Chimie, UMR CNRS 7311, Rue de Chartres-BP 6759, 45067 Orléans Cedex 2, France; tber_zahira@yahoo.com (Z.T); m.loubidi@gmail.com (M.L.); jabranejouha@gmail.com (J.J.); sabine.berteina-raboin@univ-orleans.fr (S.B.-R.);

[2] Laboratory of Molecular Chemistry, Faculty of Sciences Semlalia, Cadi Ayyad University, BP 2390, Marrakech, Morocco; i.hdoufane@gmail.com (I.H.)

[3] Department of Physiology, School of Medicine, İzmir Katip Çelebi University, Izmir, Turkey; alpero86@gmail.com (M.A.E.)

[4] Department of Physiology and Pharmacology Vittorio Erspamer, Sapienza University of Rome, P. le Aldo Moro 5, 00185 Rome, Italy; luciano.saso@uniroma1.it (L.S.)

[5] Department of Biochemistry, Faculty of Pharmacy, Ege University, 35100 Bornova, Izmir, Turkey; guliz.armagan@ege.edu.tr (G.A.)

* Correspondence: guliz.armagan@ege.edu.tr (G.A.); sabine.berteina-raboin@univ-orleans.fr (S.B.-R.);

Received: date; Accepted: date; Published: date

Experimental Section

General procedure for synthesis of compounds 1a-1i, 2a-2j, 3a-3b, 4a-4b. 2-6

*^{1}H NMR, ^{13}C NMR Spectra of compounds **4a** and **4b**, other compounds were* 7-8 *described in reference [29] RSC Adv. 2015*

pharmaceuticals

MDPI

General Procedures

9-Chloropyrido[2',1':2,3]imidazo[4,5-*c*]isoquinolin-5-amine (1a) [29]

Following the general *N*-deprotection-cyclization procedure with **S1** (187 mg, 0.49 mmol). Yellow solid (108 mg, 82 %); mp 278-279 °C; IR(neat, cm^{-1}): 765, 1084, 1273, 1324, 1427, 1625, 3177, 3300, 3444; ^{1}H NMR (400.13 MHz, DMSO-d_6): δ 7.20 (s, 2H), 7.35 (dd, J = 9.6, 2.1 Hz, 1H), 7.63 (t, J = 7.6 Hz, 1H), 7.71 (d, J = 9.6 Hz, 1H), 7.86 (t, J = 7.6 Hz, 1H), 8.43 (dd, J = 8.2, 3.1 Hz, 2H), 8.57 (d, J = 2.1 Hz, 1H); ^{13}C NMR (101 MHz, DMSO-d_6): δ 118.0, 118.4, 118.4, 120.6, 120.6, 121.7, 125.6, 125.7, 126.1, 130.8, 130.9, 134.4, 140.8, 154.6; HRMS (ESI) [M+H]$^+$: calcd for $C_{14}H_{10}ClN_4$: 269.0589, found: 269.0587.

9-Bromopyrido[2',1':2,3]imidazo[4,5-*c*]isoquinolin-5-amine (1b) [29]

Following the general *N*-deprotection-cyclization procedure with **S1** (181mg, 0.49 mmol). Yellow solid (135 mg, 88 %); mp 285-286 °C; IR (neat, cm^{-1}) : 777, 943, 1319, 1494, 1621, 3195, 3323, 3400; ^{1}H NMR (400.13 MHz, DMSO-d_6): δ 7.20 (s, 2H), 7.40 (d, J = 8.9 Hz, 1H), 7.58–7.67 (m, 2H), 7.85 (t, J = 7.4 Hz, 1H), 8.35–8.50 (m, 2H), 8.61 (s, 1H); ^{13}C NMR (101 MHz, DMSO-d_6): δ 104.8, 118.5, 118.6, 121.8, 122.7, 125.5, 125.6, 125.7, 128.1, 130.7, 130.8, 134.2, 141.0, 154.6; HRMS (ESI) [M+H]$^+$: calcd for $C_{14}H_{10}BrN_4$: 313.0083 [^{79}Br], 315.0064 [^{81}Br], found: 313.0080 [^{79}Br], 315.0062 [^{81}Br].

Methyl 5-aminopyrido[2',1':2,3]imidazo[4,5-*c*]isoquinoline-9-carboxylate (1c) [29]

Following the general *N*-deprotection-cyclization procedure with **S1** (171 mg, 0.49 mmol). Yellow solid (112 mg, 78 %); mp 296-297 °C; IR(neat, cm^{-1}): 760, 1099, 1275, 1418, 1643, 1716, 3184, 3315; ^{1}H NMR (400.13 MHz, DMSO-d_6): δ 3.93 (s, 3H), 7.31 (s, 2H), 7.58–7.75 (m, 3H), 7.87 (t, J = 7.5 Hz, 1H), 8.43 (d, J = 6.3 Hz, 2H), 9.12 (s, 1H); ^{13}C NMR (101MHz, DMSO-d_6): δ 52.4, 114.0, 117.0, 118.5, 121.8, 123.8, 125.6, 125.8, 125.9, 126.9, 130.7, 131.0, 134.8, 142.4, 154.9, 164.9; HRMS (ESI) [M+H]$^+$: calcd for $C_{16}H_{13}N_4O_2$: 293.1033, found: 293.1030.

5-Aminopyrido[2',1':2,3]imidazo[4,5-*c*]isoquinoline-9-carbonitrile (1d) [29]

Following the general *N*-deprotection-cyclization procedure with **S1** (154mg, 0.49 mmol). Yellow solid (117 mg, 92 %); mp 318-319 °C; IR (neat, cm^{-1}): 765, 1141, 1258, 1326, 1419, 1520, 1623, 2221 (CN), 3327, 3456; ^{1}H NMR (400.13 MHz, DMSO-d_6): δ 7.30 (s, 2H), 7.51 (dd, J = 9.5, 1.6 Hz, 1H), 7.66 (t, J = 7.7 Hz, 1H), 7.78 (d, J = 9.4 Hz, 1H), 7.88 (t, J = 7.5 Hz, 1H), 8.45 (t , J = 9.1 Hz, 2H), 9.17 (d, J = 1.3 Hz, 1H); ^{13}C NMR (101 MHz, DMSO-d_6): δ 95.8, 117.4, 118.2, 118.6, 121.8, 124.6, 125.7, 125.9, 126.1, 130.6, 130.7, 131.1, 134.7, 141.3, 155.1; HRMS (ESI) [M+H]$^+$: calcd for $C_{15}H_{10}N_5$: 260.0931, found: 260.0928.

9-Methylpyrido[2',1':2,3]imidazo[4,5-*c*]isoquinolin-5-amine (1e) [29]

Following the general *N*-deprotection-cyclization procedure with **S1** (149 mg, 0.49 mmol). Yellow solid (87.6 mg, 72 %); mp 247-248 °C; IR (neat, cm^{-1}): 787, 1026, 1324, 1416, 1508, 1624, 2920, 3176, 3330; ^{1}H NMR (400.13 MHz, DMSO-d_6): δ 2.37 (s, 3H), 7.03 (s, 2H), 7.20 (dd, J = 9.1, 1.7 Hz, 1H), 7.58 (t, J = 8.1 Hz, 2H), 7.83 (t, J = 7.4 Hz, 1H), 8.33 (s, 1H), 8.41 (d, J = 8.2 Hz, 2H); ^{13}C NMR (101 MHz, DMSO-d_6): δ 17.7, 116.8 , 118.1, 120.1, 120.2, 121.6, 125.1, 125.1, 125.5, 128.8, 130.5, 130.9, 134.1, 142.0, 154.0; HRMS (ESI) [M+H]$^+$: calcd for $C_{15}H_{13}N_4$: 249.1135, found: 249.1132.

9-Methoxypyrido[2',1':2,3]imidazo[4,5-*c*]isoquinolin-5-amine (1f) [29]

Following the general *N*-deprotection-cyclization procedure with **S1** (157 mg, 0.49 mmol). Yellow-green solid (118mg, 91 %); mp 236-237 °C; IR (neat, cm^{-1}): 622, 793, 1242, 1322, 1429, 1512, 1623, 3151, 3300; ^{1}H NMR (400.13 MHz, DMSO-d_6): δ 3.88 (s, 3H), 7.09 (s, 2H), 7.13 (d, J = 8.3 Hz, 1H), 7.59 (t, J = 9.8 Hz, 2H), 7.83 (t, J = 6.9 Hz, 1H), 8.00 (s, 1H), 8.40 (d, J = 7.8 Hz, 2H); ^{13}C NMR (101 MHz, DMSO-d_6): δ 55.9, 103.5, 117.9, 118.0, 120.9, 121.5, 125.1, 125.5, 125.6, 130.6, 131.1, 135.0, 140.2, 147.7, 154.0; HRMS (ESI) [M+H]$^+$: calcd for $C_{15}H_{13}N_4O$: 265.1084, found: 265.1083.

10-Methylpyrido[2',1':2,3]imidazo[4,5-*c*]isoquinolin-5-amine (1g) [29]

Following the general *N*-deprotection-cyclization procedure with **S1** (149 mg, 0.49 mmol). Yellow solid (79 mg, 65%); mp 237-238 °C; IR (neat, cm^{-1}): 761, 850, 1161, 1306, 1370, 1460, 1627, 3194, 3319; ^{1}H NMR (400.13 MHz, DMSO-d_6): δ 2.42 (s, 3H), 6.82 (d, *J* = 6.9 Hz, 1H), 7.02 (s, 2H), 7.42 (s, 1H), 7.57 (t, *J* = 7.6 Hz, 1H), 7.82 (t, *J* = 7.6 Hz, 1H), 8.39 (d, *J* = 8.4 Hz, 2H), 8.44 (d, *J* = 6.9 Hz, 1H); ^{13}C NMR (101 MHz, DMSO-d_6): δ 21.2, 113.7, 115.3, 117.9, 121.6, 122.3, 124.6, 125.0, 125.5, 130.4, 130.8, 134.2, 136.4, 143.2, 153.7; HRMS (ESI) [M+H]$^+$: calcd for $C_{15}H_{13}N_4$: 249.1135, found: 249.1132.

5-Aminopyrido[2',1':2,3]imidazo[4,5-*c*]isoquinoline-10-carbonitrile (1h) [29]

Following the general *N*-deprotection-cyclization procedure with **S1** (154 mg, 0.49 mmol). Yellow-orange solid (114 mg, 90 %); mp 340-341 °C; IR (neat, cm^{-1}): 658, 765, 1306, 1437, 1557, 1570, 1647, 2224 (CN), 3318, 3444; ^{1}H NMR (400.13 MHz, DMSO-d_6): δ 7.17 (d, *J* = 7.1 Hz, 1H), 7.46 (s, 2H), 7.67 (t, *J* = 7.7 Hz, 1H), 7.89 (t, *J* = 7.6 Hz, 1H), 8.38 (s, 1H), 8.46 (d, *J* = 7.7 Hz, 2H), 8.58 (d, *J* = 7.1 Hz,1H); ^{13}C NMR (101 MHz, DMSO-d_6): δ 105.7, 110.7, 118.4, 118.9, 122.0, 123.8, 124.3, 125.7, 126.5, 127.5, 130.5, 131.2, 135.0, 140.1, 155.7; HRMS (ESI) [M+H] $^+$: calcd for $C_{15}H_{10}N_5$: 260.0931, found: 260.0931.

Pyrido[2',1':2,3]imidazo[4,5-*c*]isoquinolin-5-amine (1i) [29]

Following the general *N*-deprotection-cyclization procedure with **S1** (170 mg, 0.49 mmol). Yellow solid (91 mg, 80 %); mp 253-255 °C (lit 254-256°C °C); IR (neat, cm^{-1}): 628, 929, 1279, 1279, 1505, 1623, 3120, 3450; ^{1}H NMR (400.13 MHz, $CDCl_3$): δ 5.26 (s, 2H), 6.87 (t, *J* = 6.7 Hz, 1H),7.30 (t, *J* = 7.9 Hz,1H), 7.58 (t, *J* = 7.7 Hz, 1H), 7.71 (d, *J* = 9.2 Hz, 1H), 7.83 (t, *J* = 7.6 Hz, 1H), 7.96 (d, *J* = 8.3 Hz, 1H), 8.58 (d, *J* = 6.8 Hz, 1H), 8.68 (d, *J* = 8.2 Hz, 1H); ^{13}C NMR (101 MHz, $CDCl_3$): δ 111.0, 118.0, 118.4, 123.0, 123.3, 124.0, 125.9, 126.4, 127.4, 131.0, 131.5, 134.1 ,144.5 ,152.5; HRMS (ESI) [M+H]$^+$: calcd for $C_{14}H_{11}N_4$: 235.0978, found: 235.0984.

***N*-(4-(Trifluoromethyl)phenyl)pyrido[2',1':2,3]imidazo[4,5-*c*]isoquinoline-5-amine (2a)** [29]

Following the general Buchwald-Hartwig cross-coupling procedure with 1-iodo-4(trifluoromethyl)benzene (62 µL, 0.426 mmol). (DCM/EA: 7:3). Yellow solid (129 mg, 80 %); mp 265-266 °C.; IR(neat, cm^{-1}): 658, 768, 1064, 1110, 1319, 1522, 1626, 3333; ^{1}H NMR (400.13 MHz, DMSO-d_6): δ 7.06 (t, *J* = 6.6 Hz, 1H), 7.46 (t, *J* = 6.2 Hz, 1H), 7.67–7.79 (m, 4H), 7.93 (t, *J* = 7.4 Hz, 1H), 8.31 (d, *J* = 8.5 Hz, 2H), 8.56 (d, *J* = 8.0 Hz, 1H), 8.74 (d, *J* = 8.4 Hz, 1H), 8.80 (d, *J* = 6.7 Hz, 1H), 9.73 (s, 1H); ^{13}C NMR (101 MHz, DMSO-d6): δ 111.5, 117.3, 119.1 (2C), 119.3, 121.0 (q, Cq, J_{CF3} = 31.8 Hz), 122.0, 123.7, 124.8 (q, Cq, J_{CF3} = 271.0 Hz), 125.0 , 125.7 (q, 2× CHar, J_{CF3} = 3.8 Hz), 126.1, 127.0, 127.2, 130.8 , 132.6, 144.2, 145.0, 147.4 (2Cq).; ^{19}F NMR (376 MHz, DMSO-d_6): δ -61.69; HRMS (ESI) [M+H]+:calcd for $C_{21}H_{14}F_3N_4$: 379.1165, found: 379.1162.

***N*-(3-Nitrophenyl)pyrido[2',1':2,3]imidazo[4,5-*c*]isoquinolin-5-amine (2b)** [29]

Following the general Buchwald-Hartwig cross-coupling procedure with 1-iodo-3-nitobenzene (106 mg, 0.426 mmol). (DCM/EA: 7:3). Red brick solid (100 mg, 66 %); mp 276-277 °C; IR (neat, cm^{-1}): 716, 821, 877, 1242, 1323, 1482, 1518, 1595, 1627, 3033, 3431; ^{1}H NMR (400.13 MHz, DMSOd_6): δ 7.05 (t, *J* = 6.7 Hz, 1H), 7.42 (t, *J* = 6.9 Hz, 1H), 7.60 (t, *J* = 8.1 Hz, 1H), 7.65–7.72 (m, 2H), 7.81 (d, *J* = 8.0 Hz, 1H), 7.88 (t, *J* = 7.5 Hz, 1H), 8.42–8.52 (m, 2H), 8.61 (d, *J* = 6.7 Hz, 1H), 8.67 (d, *J* = 8.5 Hz, 1H), 9.17 (s, 1H), 9.73 (s, 1H); ^{13}C NMR (101 MHz, DMSO-*d6*): δ 111.5, 113.4, 115.5, 117.4, 118.9, 122.0, 123.0, 124.7, 125.3, 125.9, 126.9, 127.0, 129.5, 130.7, 130.8, 132.3, 142.4, 144.1, 147.4, 147.9; HRMS (ESI) [M+H] $^+$: calcd for $C_{20}H_{14}N_5O_2$: 356.1142, found: 356.1140.

***N*-(2-(Trifluoromethyl)phenyl)pyrido[2',1':2,3]imidazo[4,5-*c*]isoquinoline-5-amine (2c)** [29]

Following the general Buchwald-Hartwig cross-coupling procedure with 1-iodo-2(trifluoromethyl)benzene (60 µL, 0.426 mmol). (DCM/EA: 7:3). Yellow solid (113 mg, 70 %); mp 180-181 °C; IR (neat, cm^{-1}): 645, 831, 1032, 1139, 1254, 1274, 1382, 1448, 1518, 1589, 1615, 2918, 3397; ^{1}H NMR (400.13 MHz, $CDCl_3$): δ 6.85 (t, *J* = 6.6 Hz, 1H), 7.13 (t, *J* = 7.5 Hz, 1H), 7.30 (dd, *J* = 9.0, 6.8 Hz, 1H), 7.56 (t, *J* = 7.9 Hz, 1H), 7.62 (t, *J* = 7.7 Hz, 1H), 7.63–7.73 (m, 3H), 7.83 (t, *J* = 7.5 Hz, 1H), 7.96 (d,

J = 8.3 Hz, 1H), 8.53 (d, J = 6.2 Hz, 1H), 8.60 (d, J = 8.4 Hz, 1H), 8.70 (d, J = 8.1 Hz, 1H); ^{13}C NMR (101 MHz, $CDCl_3$): δ 111.2, 117.8, 118.6 (q, J = 29.1 Hz), 119.6, 121.8, 122.0, 122.6

, 123.3 , 123.4 ,125.4 (q, C_q, J_{CF3} = 272.7 Hz), 126.4 (q, CH_{ar}, J = 5.5 Hz), 126.6 , 127.0 ,128.3, 130.8, 131.6, 132.6, 133.2, 139.0, 145.2, 146.9; ^{19}F NMR (376 MHz, $CDCl_3$): δ -61.57; HRMS (ESI) $[M+H]^+$: calcd for $C_{21}H_{14}F_3N_4$:379.1165, found:379.1161.

***N*-Phenylpyrido[2',1':2,3]imidazo[4,5-*c*]isoquinolin-5-amine (2d)** [29]

Following the general Buchwald-Hartwig cross-coupling procedure with iodobenzene (48 µL, 0.426 mmol). (DCM/EA: 7:3). Yellow solid (110 mg, 83 %); mp 213-214°C (lit 212-214°C); IR (neat, cm^{-1}): 661, 764, 1241, 1315, 1344, 1421, 1495, 1544, 1598, 3048, 3324; ^{1}H NMR (400.13 MHz, $CDCl_3$): δ 6.90 (t, J = 6.7 Hz, 1H), 7.11 (t, J = 7.4 Hz, 1H), 7.31–7.38 (m, 2H), 7.43 (t, J = 7.9 Hz, 2H), 7.63 (t, J = 7.6 Hz, 1H), 7.72 (d, J = 9.2 Hz, 1H), 7.83–7.86 (m, 3H), 8.09 (d, J = 8.4 Hz, 1H), 8.63 (d, J = 6.8 Hz, 1H), 8.72 (d, J = 8.1 Hz, 1H); ^{13}C NMR (101 MHz, $CDCl_3$): δ 111.2, 117.9, 119.4, 119.7 (2C), 122.7, 122.8, 123.3, 123.6, 126.3, 126.8, 127.6, 129.1 (2C), 130.7, 131.7, 133.7, 140.7, 144.9, 147.9; HRMS (m/z) $[M+H]^+$: calcd for $C_{20}H_{15}N_4$: 311.1291, found: 311.1289.

4-(Pyrido[2',1':2,3]imidazo[4,5-*c*]isoquinolin-5-ylamino)benzonitrile (2e) [29]

Following the general Buchwald-Hartwig cross-coupling procedure with 4-bromobenzonitrile (77.5 mg, 0.426 mmol). (DCM/EA: 7:3). Yellow solid (128 mg, 90 %); mp 279-280 °C; IR (neat, cm^1): 650, 728, 810, 1170, 1244, 1320, 1404, 1496, 1506, 1600, 2209 (CN), 3360; ^{1}H NMR (400.13 MHz, DMSO-d_6): δ 7.11 (t, J = 6.7 Hz, 1H), 7.50 (t, J = 6.5 Hz, 1H), 7.73–7.84 (m, 4H), 7.97 (t, J = 6.6 Hz, 1H), 8.28 (d, J = 8.7 Hz, 2H), 8.59 (d, J = 8.1 Hz, 1H), 8.74 (d, J = 8.5 Hz, 1H), 8.82 (d, J = 6.8 Hz, 1H), 9.85 (s, 1H); ^{13}C NMR (101 MHz, DMSO-d_6): δ 102.2, 111.6, 117.4, 119.0 (2C), 119.4, 119.7, 122.1, 123.8, 125.1, 126.2, 127.4, 127.4, 130.8, 131.0, 132.6 (2C), 133.0, 144.4, 145.7, 146.9; HRMS (m/z) $[M+H]^+$: calcd for $C_{21}H_{14}N_5$: 336.1244, found: 336.1241.

***N*-(Pyridin-4-yl)pyrido[2',1':2,3]imidazo[4,5-*c*]isoquinolin-5-amine (2f)** [29]

Following the general Buchwald-Hartwig cross-coupling procedure with 4-iodopyridine (87 mg, 0.426 mmol). (DCM/MeOH: 95:5).Yellow solid (91 mg, 69 %); mp 257-259 °C; IR (neat, cm^1): 731, 827, 1212, 1251, 1349, 1504, 1538, 2923, 3078, 3304; ^{1}H NMR (400.13 MHz, $CDCl_3$): δ 7.00 (t, J = 6.7 Hz, 1H), 7.42 (ddd, J = 9.0, 6.8, 1.3 Hz, 1H), 7.52 (s, 1H), 7.70 (ddd, J = 8.6, 7.1, 1.4 Hz, 1H), 7.76 – 7.80 (m, 3H), 7.90 (t, J = 7.6 Hz, 1H), 8.12 (d, J = 8.4 Hz, 1H), 8.55 (d, J = 5.3 Hz, 2H), 8.71 (d, J = 6.8 Hz, 1H), 8.78 (d, J = 8.1 Hz, 1H); ^{13}C NMR (101 MHz, $CDCl_3$): δ 111.7, 112.9 (2C), 118.1, 119.6, 122.8, 123.5, 123.6, 126.7, 127.6, 128.7, 131.1, 131.6, 145.7, 146.1, 147.6, 150.6 (2C), 151.0; HRMS (ESI) $[M+H]^+$:Masse calcd for $C_{19}H_{14}N_5$: 312.1244, found: 312.1243.

N-(Pyridin-3-yl)pyrido[2',1':2,3]imidazo[4,5-c]isoquinolin-5-amine (2g) [29]

Following the general Buchwald-Hartwig cross-coupling procedure with 3-bromopyridine (41 µL, 0.426 mmol). (DCM/MeOH: 99:1). Yellow solid (95 mg, 72 %); mp 246-247 °C; IR (neat, cm^1): 745, 930, 1251, 1348, 1415, 1521, 1582, 2930, 3080, 3314; ^{1}H NMR (400.13 MHz, $CDCl_3$): δ 6.90 (t, J = 6.7 Hz, 1H), 7.31–7. 38 (m, 2H), 7.56 (s, 1H), 7.62 (t, J = 7.6 Hz, 1H), 7.71 (d, J = 9.2 Hz, 1H), 7.83 (t, J = 7.5 Hz, 1H), 8.13 (d, J = 8.4 Hz, 1H), 8.34 (d, J = 5.8 Hz, 2H), 8.59 (d, J = 6.8Hz, 1H), 8.70 (d, J = 8.1 Hz, 1H), 9.07 (s, 1H); ^{13}C NMR (101 MHz, $CDCl_3$): δ 111.4, 117.9, 119.2, 122.8, 123.4, 123.5, 123.6, 126.4, 126.5, 127.1, 127.8, 130.8, 131.5, 133.3, 137.5, 141.7, 143.4, 145.1, 147.3. HRMS (ESI) $[M+H]^+$: calcd for $C_{19}H_{14}N_5$: 312.1244, found: 312.1243.

***N*-(Pyridin-2-yl)pyrido[2',1':2,3]imidazo[4,5-*c*]isoquinolin-5-amine (2h)** [29]

Following the general Buchwald-Hartwig cross-coupling procedure with 2-bromopyridine (41 µL, 0.426 mmol). (DCM/acetone: 9:1). Yellow solid (92 mg, 70 %); mp 175-176 °C.; IR (neat, cm^1): 756, 771, 987, 1162, 1310, 1439, 1517, 1583, 3232.;^{1}H NMR (400.13 MHz, $CDCl_3$): δ 6.94 (t, J = 6.7 Hz, 1H), 7.00 (dd, J = 7.2, 4.8 Hz, 1H), 7.36 (ddd, J = 9.2, 6.7, 1.3 Hz, 1H), 7.66 (ddd, J = 8.4, 6.9, 1.3 Hz, 1H), 7.75 (d, J = 9.2 Hz, 1H), 7.79 (ddd, J = 8.7, 7.1, 1.8 Hz, 1H), 7.87 (t, J = 7.6 Hz, 1H), 8.18 (d, J = 8.4 Hz, 1H), 8.23 (s, 1H), 8.32–8.37 (m, 1H), 8.66 (d, J = 6.8 Hz, 1H), 8.70 (d, J = 8.4 Hz, 1H), 8.73

(d, *J* = 8.2 Hz, 1H); ^{13}C NMR (101 MHz, $CDCl_3$): *δ* 111.4, 112.7 117.8, 118.0, 119.4, 123.0, 123.2, 123.4, 126.6, 127.0, 128.1, 130.9, 131.6, 133.3, 138.1, 145.2, 146.4, 148.2, 153.5; HRMS (ESI) $[M+H]^+$: calcd for $C_{19}H_{14}N_5$: 312.1244, found: 312.1243.

4-((9-Bromopyrido[2',1':2,3]imidazo[4,5-*c*]isoquinolin-5-yl)amino)benzonitrile (2i) [29]

Following the general Buchwald-Hartwig cross-coupling procedure with 4-bromobenzonitrile (77.5 mg, 0.426 mmol). (DCM/EA: 7:3).Yellow solid (127 mg, 72 %); mp 320-321 °C; IR (neat, cm^{-1}): 707, 769, 833, 1241, 1407, 1517, 1595, 2219 (CN), 3340; ^{1}H NMR (400.13 MHz, DMSO-*d*$_6$): *δ* 7.58 (dd, *J* = 9.5, 1.9 Hz, 1H). 7.75 (d, *J* = 9.6 Hz, 1H), 7.79 (t, *J* = 7.6 Hz, 1H), 7.83 (d, *J* = 8.7 Hz, 2H), 7.98 (t, *J* = 7.6 Hz, 1H), 8.30 (d, *J* = 8.8 Hz, 2H), 8.57 (d, *J* = 8.0 Hz, 1H), 8.75 (d, *J* = 8.5 Hz, 1H), 8.97 (d, *J* = 1.9 Hz, 1H), 9.87 (s, 1H); ^{13}C NMR (101 MHz, DMSO-*d*$_6$): *δ* 102.5, 105.5, 118.6, 119.3 (2C), 119.6, 119.7, 122.1, 123.6, 125.1, 126.5, 127.7, 130.1, 130.7, 131.3, 132.5, 133.0 (2C), 142.6, 145.4, 147.5; HRMS (m/z) $[M+H]^+$: calcd for $C_{21}H_{13}BrN_5$: 414.0349 [^{79}Br], 416.0331 [^{81}Br], found: 414.0350 [^{79}Br], 414.0349 [^{81}Br].

4-((9-Chloropyrido[2',1':2,3]imidazo[4,5-*c*]isoquinolin-5-yl)amino)benzonitrile (2j) [29]

Following the general Buchwald-Hartwig cross-coupling procedure with 4-bromobenzonitrile (77.5mg, 0.426 mmol). (DCM/EA: 7:3). Yellow solid (116mg, 74 %); mp 319-320 °C.; IR (neat, cm^1): 661, 765, 1089, 1173, 1244, 1511, 2212 (CN), 3376; ^{1}H NMR (400.13 MHz, DMSO-*d*$_6$): *δ* 7.49 (d, *J* = 8.2 Hz, 1H), 7.76–7.83 (m, 4H), 7.96 (t, *J* = 7.4 Hz, 1H), 8.29 (d, *J* = 8.3 Hz, 2H), 8.54 (d, *J* = 8.1 Hz, 1H), 8.72 (d, *J* = 8.5 Hz, 1H), 8.88 (s, 1H), 9.83 (s, 1H); ^{13}C NMR (101 MHz, DMSO-*d*$_6$): *δ* 102.5, 118.3, 118.6, 119.3 (2C), 119.5, 119.7, 121.6, 122.1, 125.1, 126.4, 127.9, 130.7, 131.2, 132.6, 133.0 (2C), 142.5, 145.4, 147.5, 147,7; HRMS (m/z) $[M+H]^+$: calcd for $C_{21}H_{13}ClN_5$: 370.0854, found: 370.0852.

9-Chloropyridazino[6',1':2,3]imidazo[4,5-*c*]isoquinolin-5-amine (3a) [29]

Following the general *N*-deprotection-cyclization procedure with S2 (159 mg, 0.49 mmol). Yellow orange solid (100 mg, 89 %); mp 299-300 °C; IR (neat, cm^{-1}): 708, 799, 1086, 1099, 1250, 1305, 1423, 1480, 1507, 3024, 3106, 3281; ^{1}H NMR (400.13 MHz, DMSO-*d*$_6$): *δ* 7.33 (d, *J* = 9.5 Hz, 1H), 7.47 (s, 2H), 7.67 (t, *J* = 7.6 Hz, 1H), 7.89 (t, *J* = 7.5 Hz, 1H), 8.24 (d, *J* = 9.5 Hz, 1H), 8.45 (t, *J* = 8.3 Hz, 2H). ^{13}C NMR (101 MHz, DMSO-*d*$_6$): *δ* 118.6, 118.8, 121.6, 125.6, 126.1, 126.4, 127.9, 130.7, 131.2, 135.8, 135.8, 145.3, 156.0; HRMS (ESI) $[M+H]^+$: calcd for $C_{13}H_9ClN_5$: 270.0541, found: 270.0540.

4-((9-Chloropyridazino[6',1':2,3]imidazo[4,5-c]isoquinolin-5-yl)amino)benzonitrile (3b) [29]

Following the general Buchwald-Hartwig cross-coupling procedure with 4-bromobenzonitrile (77.5mg, 0.426 mmol). (DCM/EA: 7:3). Yellow solid (116mg, 74 %); mp: 319-320 °C.; IR (neat, cm^1): 661, 765, 1089, 1173, 1244, 1511, 2212 (CN), 3376; ^{1}H NMR (400.13 MHz, DMSO-*d*$_6$): *δ* 7.49 (d, *J* = 8.2 Hz, 1H), 7.76–7.83 (m, 4H), 7.96 (t, *J* = 7.4 Hz, 1H), 8.29 (d, *J* = 8.3 Hz, 2H), 8.54 (d, *J* = 8.1 Hz, 1H), 8.72 (d, *J* = 8.5 Hz, 1H), 8.88 (s, 1H), 9.83 (s, 1H); ^{13}C NMR (101 MHz, DMSO-*d*$_6$): *δ* 102.5, 118.3, 118.6, 119.3 (2C), 119.5, 119.7, 121.6, 122.1, 125.1, 126.4, 127.9, 130.7, 131.2, 132.6, 133.0 (2C), 142.5, 145.4, 147.5, 147,7; HRMS (m/z) $[M+H]^+$: calcd for $C_{21}H_{13}ClN_5$: 370.0854, found: 370.0852.

6-Cyclohexylpyrido[2',1':2,3]imidazo[4,5-c]isoquinolin-5(6H)-imine (4a)

Following the general *N*-deprotection-cyclization procedure (84,0 mg, 0,265 mmol), DCM/MeOH (95/5), Yellow solid (84mg, 80 %); mp: 172-173 °C, IR (neat, cm^{-1}): 671,724, 797, 1128, 1186, 1448, 1631, 1669, 3359, 3439. NMR ^{1}H (DMSO-*d*$_6$, 400,13 MHz): δ 1.44 – 1.70 (m, 4H**)**, 1. 91 – 1.95 (m, 2H), 2.05 – 2.09 (m, 2H), 2.68 – 2.78 (m, 2H), 4.75 – 4.81 (m, 2H), 7.18 (t, *J* = 6.8 Hz, 1H), 7.44 (t, *J* = 6.8 Hz, 1H), 7.73 (t, , *J* = 7.9 Hz, 1H), 7.79 (d, *J* =6.8 Hz, 1H), 8.01 (t, , *J* = 7.9 Hz, 1H), 8.28 (d, *J* = 6.8 Hz, 1H), 8.41 (d, *J* = 7.9 Hz, 1H), 8.71 (d, 1H, *J* = 7.9 Hz), 9.33 (s, 1H); NMR ^{13}C (DMSO-*d*$_6$, 101 MHz): *δ* 24.1, 25.9 (2C), 28.4 (2C), 63.9, 114.0, 116.2, 118.7, 119.2, 122.7, 125.3, 125.6, 126.1, 127.1, 128.5, 131.4, 134.8, 143.8, 157.2; HRMS (m/z) $[M+H]^+$: calcd for $C_{20}H_{21}N_4$: 317,1761, found: 317, 1760.

6-Benzylpyrido[2',1':2,3]imidazo[4,5-*c*]isoquinolin-5(6*H*)-imine (4b)

Following the general *N*-deprotection-cyclization procedure(83,0 mg, 0,256 mmol DCM/MeOH (99/1). Yellow solid (83mg, 78 %); mp: 174-175 °C; IR (neat, cm^{-1}): 663, 730, 898, 950, 1123, 1328, 1253, 1520, 1598,

1616, 3307; NMR ^{1}H (DMSO-d_6, 400,13 MHz): δ 5.96 (s, 2H), 6.65 (t, J = 6.9 Hz, 1H), 7.03 (t, J = 6.9 Hz, 1H), 7.24 (t, J = 7.9 Hz, 1H), 7.27 – 7.37 (m, 4H), 7.56 – 7.46 (m, 2H), 7.72 (t, J = 7.9 Hz, 1H), 8.19 (d, J = 6.9 Hz, 1H), 8.22 (d, J = 7.9 Hz, 1H), 8.38 (d, J = 7.9 Hz, 1H), 8.60 (s, 1H); NMR ^{13}C (DMSO-d_6, 101 MHz): δ 47.4, 112.0, 117.9, 121.8, 122.8, 122.9, 123.5, 123.7, 125.6 (2C), 126.1, 127.0, 127.0, 127.2, 128.8 (2C), 130.0, 131.6, 137.5, 141.5, 156.9; HRMS (m/z) HRMS (m/z) $[M+H]^+$: calcd for: $[M+H]^+$, $C_{21}H_{17}N_4$: 325.1448, found: 325.1149

6-cyclohexylpyrido[2',1':2,3]imidazo[4,5-c]isoquinolin-5(6H)-imine (4a)

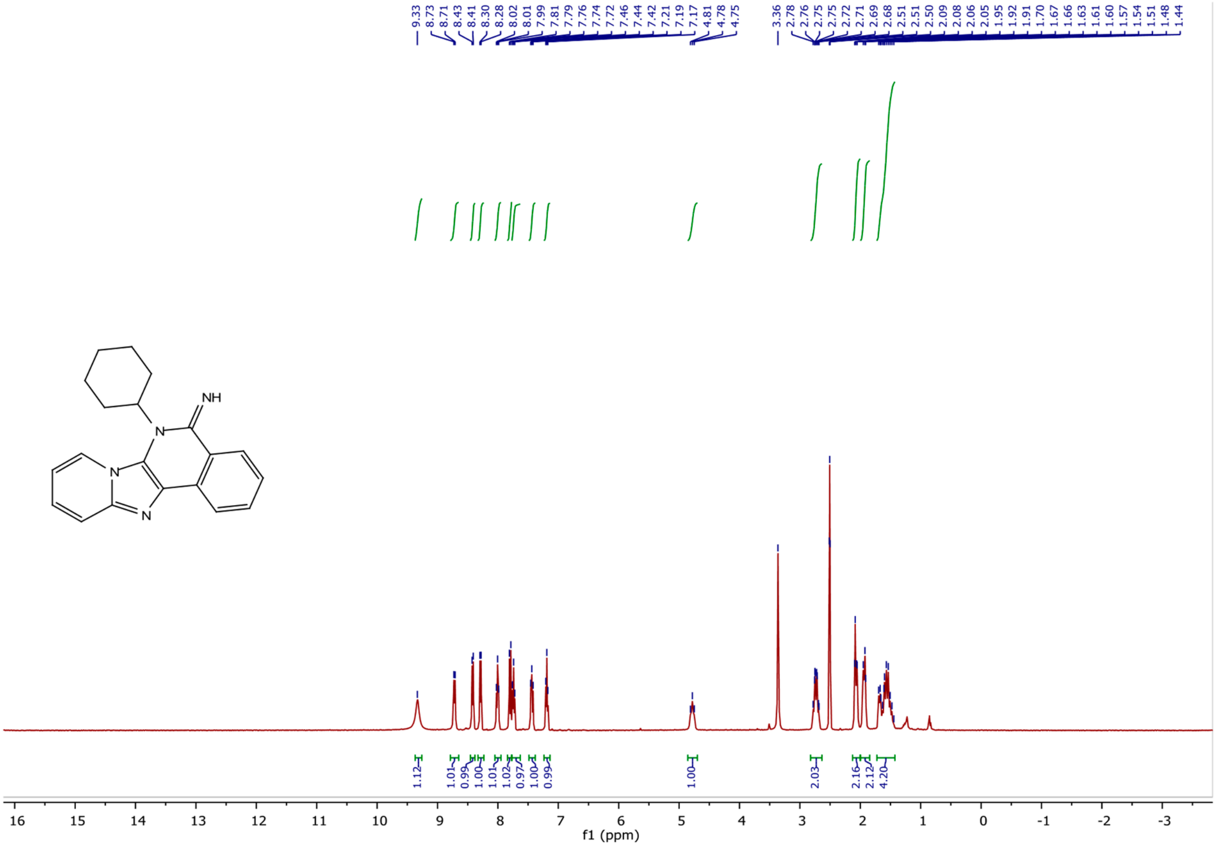
9.33
8.73
8.71
8.43
8.41
8.30
8.28
8.02
8.01
7.99
7.81
7.79
7.76
7.74
7.72
7.46
7.44
7.42
7.21
7.19
7.17
4.81
4.78
4.75
3.36
2.78
2.76
2.75
2.75
2.72
2.71
2.69
2.68
2.51
2.51
2.50
2.09
2.08
2.06
2.05
1.95
1.92
1.91
1.70
1.67
1.66
1.63
1.61
1.60
1.57
1.54
1.51
1.48
1.44
NH
N
N
N
1.12
1.01
0.99
1.00
1.01
1.02
0.97
1.00
0.99
1.00
2.03
2.16
2.12
4.20
f1 (ppm)

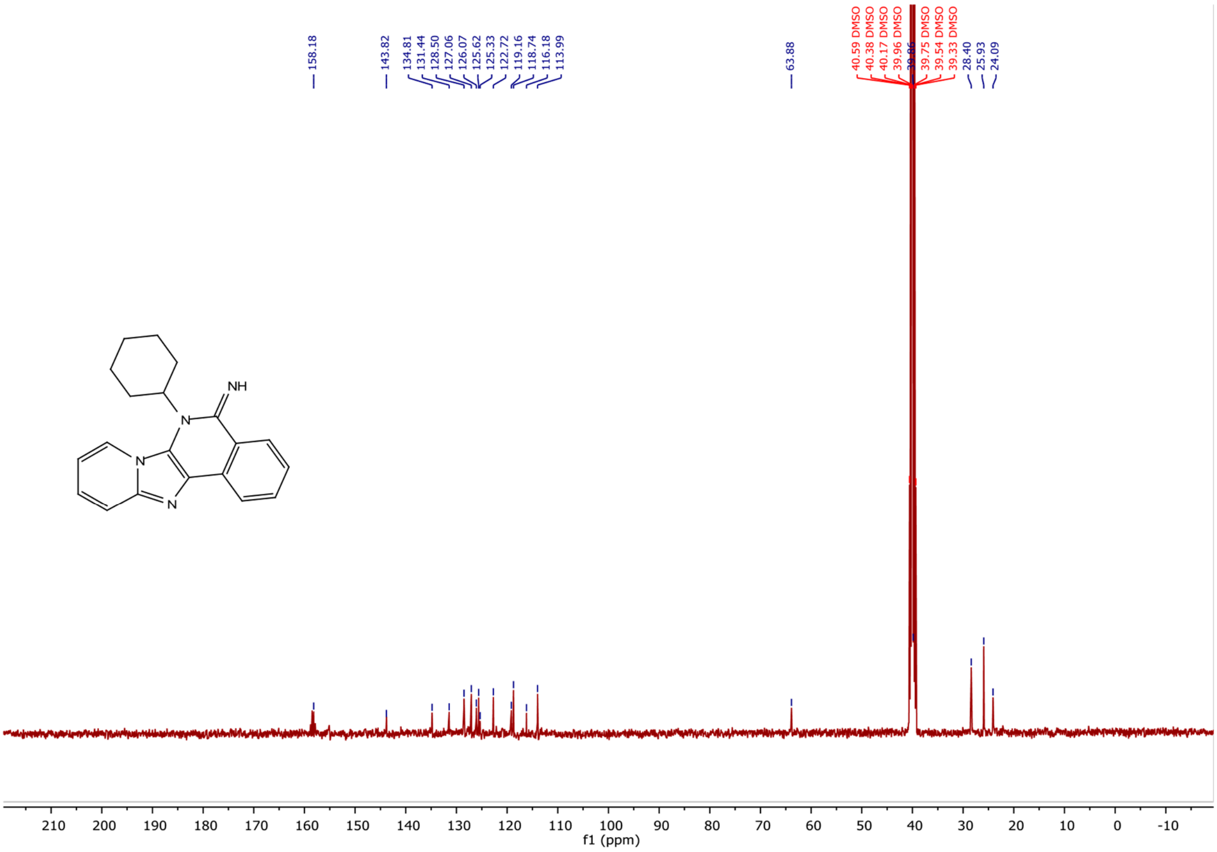
158.18
143.82
134.81
131.44
128.50
127.06
126.07
125.62
125.33
122.72
119.16
118.74
116.18
113.99
63.88
40.59 DMSO
40.38 DMSO
40.17 DMSO
39.96 DMSO
39.75 DMSO
39.54 DMSO
39.33 DMSO
28.40
25.93
24.09
NH
N
N
N
f1 (ppm)

pharmaceuticals

MDPI

6-benzylpyrido[2',1':2,3]imidazo[4,5-c]isoquinolin-5(6H)-imine (4b)

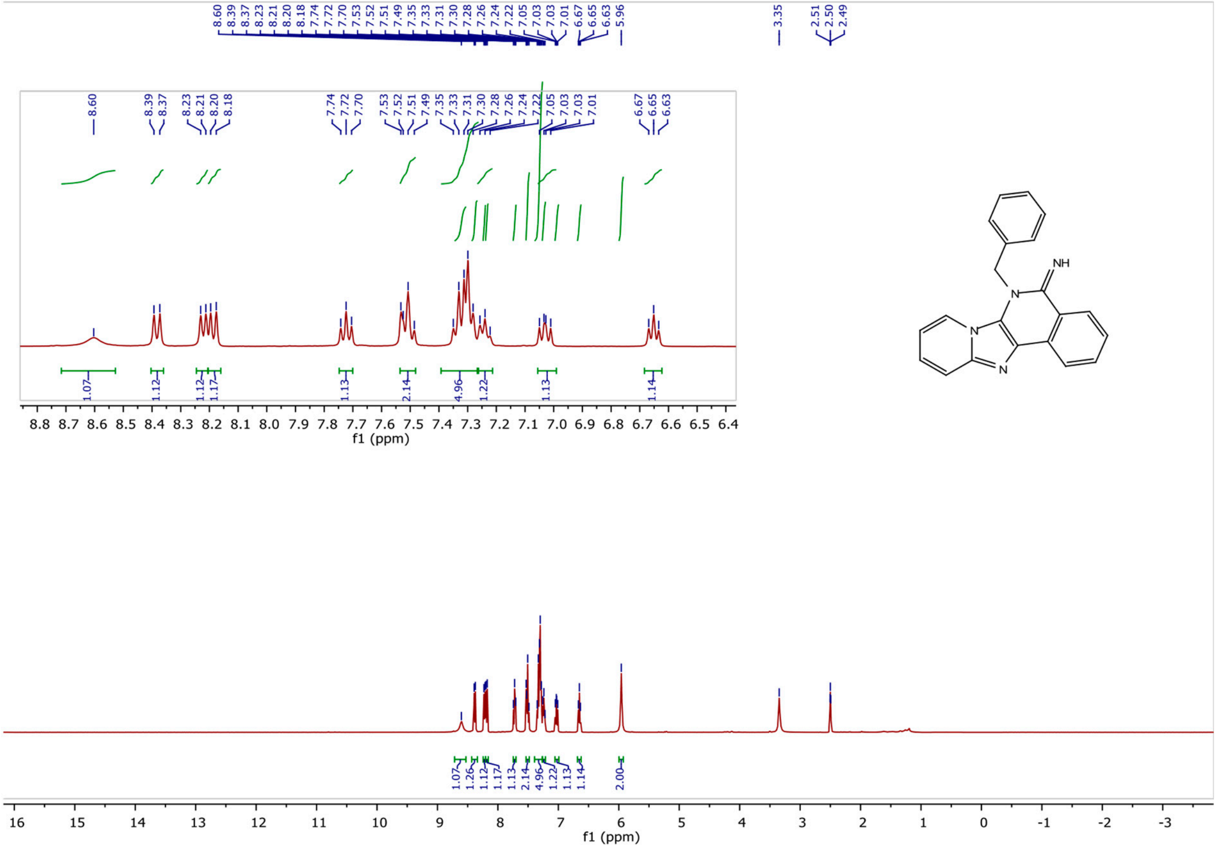

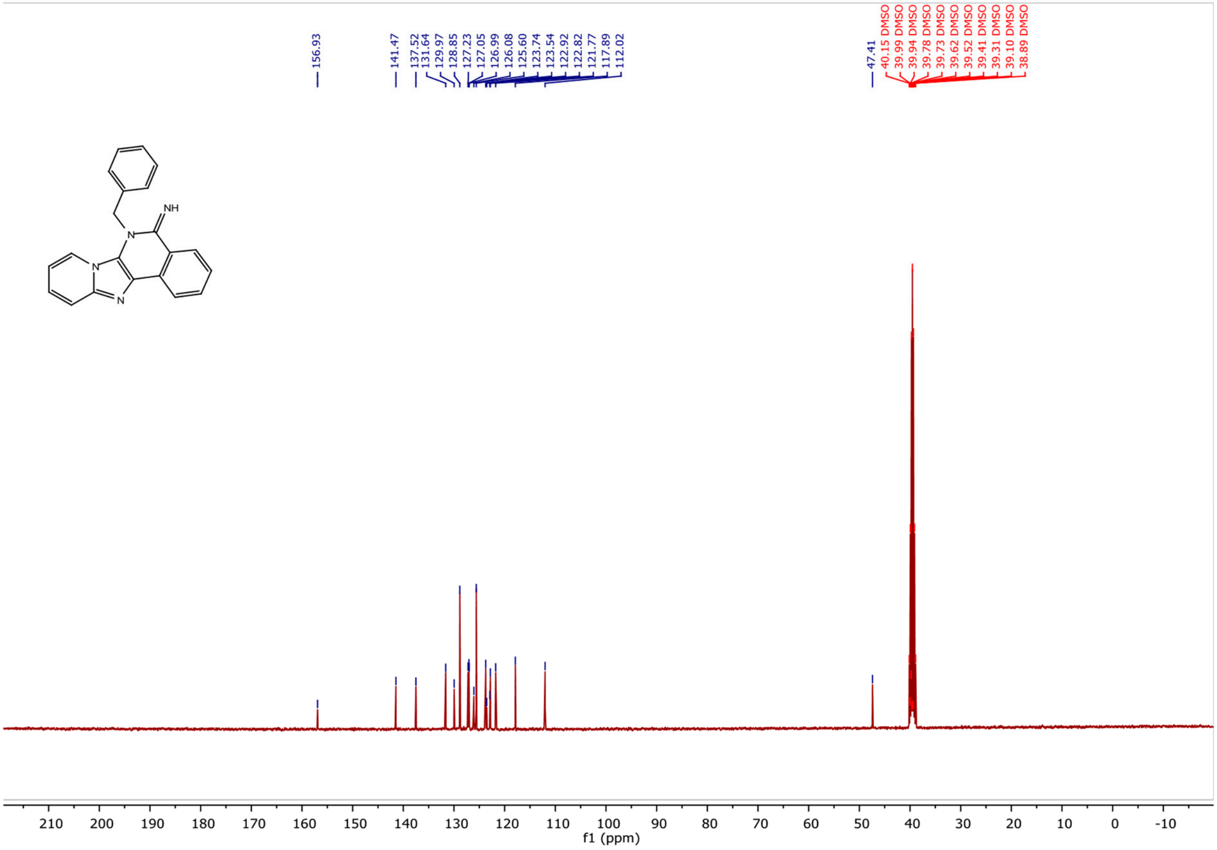

Supplement: Supplementary file 1 [file pharmaceuticals-14-00750-s001.zip › pharmaceuticals-1282000-supplementary.pdf]
